# Supplementary material for: A horizon scan of future threats and opportunities for pollinators and pollination
Source: PeerJ. 2016 Aug 9;4:e2249. doi: 10.7717/peerj.2249 (PMC4991895; doi:10.7717/peerj.2249)
Supplement: Table S1 [file peerj-04-2249-s001.docx]

Table S1. The initial list of potential issues identified by the horizon-scanning process. Issues with the same number were grouped together, based on similarity, for voting.

| **#** | **Title** | **Summary** | **References** | |
| --- | --- | --- | --- | --- |
| 1 | Sulfoximine, a novel systemic class of insecticides | Sulfoximines are a new class of neuroactive insecticides. Like the neonicotinoids, which are now widely suspected as contributors to honey bee colony losses and wild bee population declines (Goulson *et al.* 2015), sulfoximines act on insect nicotinic receptors (Sparks *et al.* 2013). Also similar to neonicotinoids, they are water-soluble systemic insecticides that are taken up by plants and expressed throughout the tissues, including nectar and pollen that may be ingested by foraging pollinators. However, their chemistry and specific mode of action differs from neonicotinoids, such that these compounds are effective against target pests (mostly sap-sucking and root-feeding insects) that have evolved resistance to neonicotinoids or other class of insecticides (Sparks *et al.* 2013). The first member of this new insecticide family is Sufloxaflor (Dow Agrichemical Company). Why Sulfoxaflor, and more importantly, sulfoximines as an insecticide class, constitute a horizon scan issue for pollinators is because: (1) this new chemistry is being pushed by chemical companies as a replacement for neonicotinoids against sap-sucking insects <http://newsroom.dowagro.com/press-release/dow-agrosciences-receives-us-epa-registration-sulfoxaflor>, since resistance is developing (Bass *et al.* 2015); (2) neonicotinoid uptake was relatively rapid and has led to widespread use globally (Simon-Delso *et al.* 2014); if sulfoximines were to be taken up as a replacement for neonicotinoids, they could ultimately impact a similarly large land area and number of crops; (3) like neonicotinoids, sulfoximines are likely to have important sub-lethal as well as lethal effects; while the sub-lethal effects of neonicotinoids are just beginning to be appreciated (e.g., Rundlöf *et al.* 2015), no information is yet available on sublethal effects of sulfoximines (European Food Safety Authority 2014)  **Further information:**  Sulfoxaflor was first registered for use by Dow Agrichemical Company in South Korea in 2011 and was subsequently registered in the USA and China in 2013, in Canada and the European Union in 2015, and also in Australia, Panama, Vietnam, Indonesia and Guatemala [1], and is used in several trademark products [1]. As yet, all formulations are spray applications but a seed treatment, as well as new related chemicals within this family, could be developed in the future. This relatively rapid deployment in a variety of countries could signal the beginning of an exponential uptake, as occurred with neonicotinoid pesticides, which currently occupy approximately one-quarter of the global pesticide market following their discovery in the late 1980’s (Simon-Delso *et al.* 2014). Like neonicotinoids, sulfoximines can have broad applications in agriculture, horticulture and veterinary medicine. One of the largest current uses of neonicotinoids is as a prophylactic seed treatment on canola, corn, sunflower, soy and other crops (Furlan & Kreutzweiser 2014), now making up, for example, 100% of the corn acreage in the United States and 70% of the soy (Douglas & Tooker 2015). Given consolidation in the seed industry and its links to chemical companies (Howard 2009), obtaining corn and soy seed that is not treated with neonicotinoids in the US is quite difficult (Douglas & Tooker 2015). Possibly due to such widespread use, concerns about resistance are beginning to develop (Simon-Delso *et al.* 2014; Bass *et al.* 2015). It is well-known that as resistance develops and secondary pests emerge, chemical companies seek to develop and market alternative chemistries; given the lack of chemical cross-resistance with neonicotinoids (Sparks *et al.* 2013), sufloximines seem like a likely candidate for replacement.  Similar to neonicotinoids, registration for Sulfoxaflor in the United States has proceeded, despite sufficient knowledge of what constitutes field-realistic exposure rates for foraging bees, and how prolonged sub-lethal exposure affects individuals, colonies and populations, as documented in the record of public comment and response by the Environmental Protection Agency ([file:///Users/ck/Downloads/EPA-HQ-OPP-2010-0889-0397.pdf](file:///C:\Users\ck\Downloads\EPA-HQ-OPP-2010-0889-0397.pdf)). Evidence is only recently accumulating to show that field-level exposure and/or field-realistic doses of neonicotinoids have negative effects on wild pollinators (Whitehorn *et al.* 2012; Rundlöf *et al.* 2015), but huge research gaps still exist for these compounds (Goulson *et al.* 2015). However by comparison we know almost nothing about the effects of sufloximines on pollinators [1]. Considered high risk to foraging honeybees and other non-target arthropods based on the few available semi-field trials (European Food Safety Authority 2014) [4] as yet there is no knowledge of how this compound would affect honey bee larvae, of sub-lethal effects, or effects on wild bees (European Food Safety Authority 2014).  As a counter to this scenario of broad future adoption of systemic sulfoximines, it could be that the recognition of problems with neonicotinoids and the struggles against it will mean that organizations will campaign against sulfoximines sooner, (as is already happening but with little success: <http://www.beyondpesticides.org/dailynewsblog/2013/05/epa-green-lights-new-pesticide-highly-toxic-to-bees-dismisses-concerns/>; <http://www.centerforfoodsafety.org/files/cfs_may2014_sulfoxaflor_comments_17155.pdf>, etc), to prevent its broad spread. | Bass, C., Denholm, I., Williamson, M.S. & Nauen, R. (2015) The global status of insect resistance to neonicotinoid insecticides. *Pesticide Biochemistry and Physiology*, 121, 78–87.  European Food Safety Authority. (2014) Conclusion on the peer review of the pesticide risk assessment of the active substance sulfoxaflor. *European Food Safety Authority Journal*, 12, 3692.  Goulson, D., Nicholls, E., Botías, C. & Rotheray, E.L. (2015) Bee declines driven by combined stress from parasites , pesticides , and lack of flowers. *Science*, 347, 1435.  Rundlöf, M., Andersson, G.K.S., Bommarco, R., Fries, I., Hederström, V., Herbertsson, L., Jonsson, O., Klatt, B.K., Pedersen, T.R., Yourstone, J. & Smith, H.G. (2015) Seed coating with a neonicotinoid insecticide negatively affects wild bees. *Nature*, 521, 77–80.  Simon-Delso, N., Amaral-Rogers, V., Belzunces, L.P., Bonmatin, J.M., Chagnon, M., Downs, C., Furlan, L., Gibbons, D.W., Giorio, C., Girolami, V., Goulson, D., Kreutzweiser, D.P., Krupke, C.H., Liess, M., Long, E., McField, M., Mineau, P., Mitchell, E. a. D., Morrissey, C. a., Noome, D. a., Pisa, L., Settele, J., Stark, J.D., Tapparo, A., Van Dyck, H., Van Praagh, J., Van der Sluijs, J.P., Whitehorn, P.R. & Wiemers, M. (2014) Systemic insecticides (neonicotinoids and fipronil): trends, uses, mode of action and metabolites. *Environmental Science and Pollution Research*, 22, 5–34.  Sparks, T.C., Watson, G.B., Loso, M.R., Geng, C., Babcock, J.M. & Thomas, J.D. (2013) Sulfoxaflor and the sulfoximine insecticides: Chemistry, mode of action and basis for efficacy on resistant insects. *Pesticide Biochemistry and Physiology*, 107, 1–7  **Further information notes**  [](<http://www.epa.gov/oppfead1/cb/csb_page/updates/2013/sulfoxaflor-decision.html>; <http://news.agropages.com/News/NewsDetail---9776.htm>; <http://www.hc-sc.gc.ca/cps-spc/pubs/pest/_decisions/rd2015-09/index-eng.php>; <http://news.agropages.com/News/NewsDetail---13701.htm>; <http://newsroom.dowagro.com/press-release/dow-agrosciences-receives-us-epa-registration-sulfoxaflor>)  [2] Closer^TM^, Transform^TM^  [3] A search on ((sulfoxaflor OR sulfoximine) AND (bee or Apis or honeybee)) on Web of Science yielded 6 results, or which 3 were patents and one was not relevant. The other two are cited.  [4] But curiously, not considered high risk to the US, Canadian and Australian joint commission of pesticide regulators that reviewed the same evidence and then registered the chemical. | |
| 1 | New pesticides | History suggests that we have gone through repeated cycles of approving new pesticides and then subsequently, often decades later, recognizing that they do significant harm to the environment and withdrawing them (e.g. organochlorides, organophosphates and, arguably, neonicotinoids). Independent scientists usually do not know what pesticides are coming through the regulatory system until they are approved for use. Hence we are entirely reliant on the regulatory bodies doing their job effectively, yet history shows that in the past they have failed to do so. One clear failing of the current system is that it is unable to properly assess the risks posed by chronic exposure or exposure to pesticide mixes. Recently, two new insecticides have been approved for use: sulfoxaflor and flupyradifurone. Both are chemically similar to neonicotinoids (although their manufacturers stress that they are different), and are systemic so are likely to get into pollen and nectar of flowering crops (Babcock et al. 2010; Nauen et al. 2015). The safety of these chemicals for pollinators will only be properly understood when they have been in use for a decade or more, which is rather late to detect any adverse effects. | <https://www.federalregister.gov/articles/2015/01/23/2015-01013/flupyradifurone-pesticide-tolerances>  <http://www.takepart.com/article/2015/05/07/epa-approves-new-pesticide-environmentalists-say-threatens-bees>  Babcock et al. 2010: <http://onlinelibrary.wiley.com/doi/10.1002/ps.2069/pdf>  Nauen et al. 2015: <http://www.ncbi.nlm.nih.gov/pubmed/25351824> | |
| 1 | Novel Pesticides: new threats | High-profile evidence for the negative impact of neonicotinoids (neonics) on honey bees (*Apis mellifera*; Henry et al. 2012) and bumble bees (*Bombus*; Whitehorn et al. 2012) led the EU to ban the use of two of this class of compound for two years (December 2013-December 2015: <http://ec.europa.eu/food/archive/animal/liveanimals/bees/neonicotinoids_en.htm>). Field-realistic tests have demonstrated that neonics harm bumble bees, though impacts on honey bees are not readily discernible (Rundlöf et al. 2015). In addition, insect pests inevitably evolve resistance to synthetic pesticides (e.g. whitefly resistance to neonics: <http://www.bioone.org/doi/abs/10.1603/EC12414>). Companies producing plant protection compounds will inevitably develop novel insecticides, and probably already sit on a large number of potential compounds. As classes of pesticide are banned or as pests evolve resistance to them, new and potentially more insidious insecticides may be introduced that unwittingly harm pollinators to an even greater extent than those currently in use. More specifically, a ban on neonics may herald introduction of novel pesticides more insidious for bees.  Considerably more thorough assessment of pesticides by truly independent regulatory authorities, and coordination of these authorities across countries, provides one solution so that indirect as well as direct effects of pesticides to non-target organisms are assessed. Indirect effects should include interactions with other pesticides and pathogens. | Henry M, Béguin M, Requier F*, et al.* (2012) A common pesticide decreases foraging success and survival in honey bees. *Science* 336, 348-350.  Rundlöf M, Andersson GKS, Bommarco R*, et al.* (2015) Seed coating with a neonicotinoid insecticide negatively affects wild bees. *Nature* 521, 77–80.  Whitehorn PR, O’Connor S, Wackers FL, Goulson D (2012) Neonicotinoid pesticide reduces bumble bee colony growth and queen production. *Science* 336, 351-352 | |
| 1 | New PPPs (Plant Protection Products) | The EU has recently approved a new neonicotinoid pesticide (Sulfoxaflor) for use (European Commission 2015), despite EFSA concluding there were data gaps concerning potential risks of field use to non-target organisms (including bees and small mammals) (EFSA 2015). in most cases, we lack data on field-level exposure (due to little knowledge on frequency and magnitude of PPP use, residues in the soil and uptake by wild plants, and the effects of highly fluctuating land-use change), as well as proper understanding of non-target impacts on all but a few experimental model species (Rundlöf et al. 2015), and knowledge of long-term impacts of existing PPPs. Therefore it seems inappropriate to introduce new pesticides in the same chemical class as those that have been shown to be detrimental. Other classes of PPPs are likely to be developed and we are lacking appropriate assessment of sub-lethal non-target impacts on an appropriate range of organisms. Most toxicity testing only uses honeybees as a model – they react differently to potentially toxic chemicals in their diet compared with other bees (Tiedeken et al. in prep). Furthermore, the use of these chemicals in domestic situations (in gardens and as flea treatments for pets for example) and the consequent exposure to pollinators is unknown. | EFSA (European Food Safety Authority), 2014. Conclusion on the peer review of the pesticide risk assessment of the active substance sulfoxaflor. *EFSA Journal* 2014; 12(5): 3692, 170 pp. doi:10.2903/j.efsa.2014.3692 <http://www.efsa.europa.eu/en/efsajournal/pub/3692>  European Commission (2015) COMMISSION IMPLEMENTING REGULATION (EU) 2015/1295 of 27 July 2015 approving the active substance sulfoxaflor, in accordance with Regulation (EC) No 1107/2009 of the European Parliament and of the Council concerning the placing of plant protection products on the market, and amending the Annex to Commission Implementing Regulation (EU) No 540/2011 <http://eur-lex.europa.eu/legal-content/EN/TXT/PDF/?uri=CELEX:32015R1295&from=EN>  Rundlöf, M., Andersson, G.K.S., Bommarco, R., Fries, I., Hederström, V., Herbertsson, L., Jonsson, O., Klatt, B.K., Pedersen, T.R., Yourstone, J., Smith, H.G., 2015. Seed coating with a neonicotinoid insecticide negatively affects wild bees. *Nature* 521, 77-80.  Tiedeken, E.J., Egan, P.A., Stevenson, P.C., Wright, G.A., Brown, M.J.F., Power, E.F., Farrell, I., Matthews, S.M., Stout, J.C. (in prep). Nectar chemistry modulates the impact of an invasive plant on native pollinators. *Functional Ecology* (accepted subject to revisions) | |
| 2 | The effect of chemical use on pollinators in non-agricultural settings | Whilst the effect of chemicals on pollinators in agricultural settings has received much attention from scientists, policymakers and the public in recent years (Godfray *et al.* 2014, Rundlöf *et al.* 2015) far less consideration has been given to the potential impacts of insecticides on pollinators in non-crop settings. A wide range of chemicals are applied to grass lawns and to garden plants to control invertebrate pests and weeds. Several recent studies indicate that some of these chemicals can negatively affect pollinating insects. Larson *et al.* (2013) found that neonicotinoid insecticide use in lawns can negatively affect bumblebee colonies if applied to blooming plants and a study using citizen science data from gardens in France found a negative correlation between butterfly and bumblebee abundance and use of insecticides and herbicides (Muratet & Fontaine 2015). Muratet & Fontaine (2015) also found that the negative effect of insecticides was stronger in highly urbanised areas. Given that gardens and lawns comprise large areas (Loram *et al.* 2007, 2008) and that chemicals are widely used by professional and domestic gardeners, further consideration should be given to the potentially negative effect of garden chemicals on pollinators as well as to alternative pest control measures that gardeners can employ. | Godfray HCJ, Blacquiere T, Field LM, Hails RS, Petrokofsky G, Potts SG, Raine NE, Vanbergen AJ, & AR McLean (2014) A restatement of the natural science evidence base concerning neonicotinoid insecticides and insect pollinators. *Proc. R. Soc. B* **281**: 20140558.  Larson, JL, Redmond CT & Potter DA (2013) Assessing Insecticide Hazard to Bumble Bees Foraging on Flowering Weeds in Treated Lawns. *PLoS ONE* **8** .  Loram A, Tratalos J, Warren P & Gaston K. (2007) Urban domestic gardens (X): the extent & structure of the resource in five major cities. *Landscape Ecol* **22**:601–615  Loram A, Warren P & Gaston K. (2008) Urban Domestic Gardens (XIV): The Characteristics of Gardens in Five Cities. *Environmental Management* **42**:361-376.  Muratet A & Fontaine B (2015) Contrasting impacts of pesticides on butterflies and bumblebees in private gardens in France. *Biological Conservation* **182**: 148–154.  Rundlof M, Andersson GKS, Bommarco R, Fries I, Hederstrom V, Herbertsson L, Jonsson O, Klatt BK, Pedersen TR, Yourstone J & Smith HG (2015) Seed coating with a neonicotinoid insecticide negatively affects wild bees. *Nature* **521**: 77-80 | |
| 3 | Increasing use of fungicides | Fungicides have impacts on pollinators, especially when exposed to insecticides at the same time (Pettis et al. 2013; Bernuer et al., 2015; Zubord et al. 2014). Questions exist about whether fungicide risk assessment is good enough to protect pollinators and other wildlife (Zubord et al. 2014). Three possible trends could lead to a general increase in fungicide use: increasing fungicide resistance (Southeast Farm Press 2015), increasing production of insect-pollinated crops such as soft fruits, and climate change leading to warmer wetter conditions. | Pettis et al., 2013 "Crop Pollination Exposes Honey Bees to Pesticides Which Alters Their Susceptibility to the Gut Pathogen *Nosema ceranae*." PLoS One  Bernauer et al., 2015. "Colonies of Bumble Bees (Bombus impatiens) Produce Fewer Workers, Less Bee Biomass, and Have Smaller Mother Queens Following Fungicide Exposure." *Insects*  Zubrod et al., 2014. "Does the Current Fungicide Risk Assessment Provide Sufficient Protection for Key Drivers in Aquatic Ecosystem Functioning?" Envt. Science & Technology. http://pubs.acs.org/doi/abs/10.1021/es5050453  Southeast Farm Press, 2015. "It matters: 6 steps to avoid fungicide resistance now." http://southeastfarmpress.com/soybeans/it-matters-6-steps-avoid-fungicide-resistance-now | |
| 4 | Aluminium | Aluminium has recently been discovered in bumblebee pupae from colonies in S England at levels that would be highly toxic to humans (Exley et al. 2015). It has previously been found at moderately high concentrations in honeybees (van der Steen & de Kraker 2012). Bumblebee colonies with higher levels of aluminium had significantly smaller pupae, though no other adverse effects were found. It is not clear where this contamination came from, or whether aluminium naturally occurs at high concentrations in bees. An experiment is needed to test whether aluminium is harmful to bees at the observed concentrations. | Exley C, Rotheray E, Goulson D. 2015. Bumblebee pupae contain high levels of aluminium. *PlosONE* 10: e0127665  van der Steen JJM, de Kraker J (2012) Spatial and temporal variation of metal concentrations in adult honeybees (*Apis mellifera* L.). *Environ Monit Assess* 184: 4119-4126 | |
| 5 | Potential non-target effects of nanoparticle pesticides on crop visiting insect pollinators | Nanotechnology – or the manipulation of matter with at least one dimension sized from 1 to 100 nanometers (National Nanotechnology Initiative 2014) – is a rapidly growing field with a wide variety of potential applications. Many nanoparticles are already in use (Vance et al. 2015), with many more products containing this technology on the horizon. In agriculture, nanotechnology holds the promise of improving productivity and reducing pesticide use (Sekhon 2014). Many nanoparticle pesticides that have been microencapsulated for delivery have been shown to be effective against various insect pests (Green et al. 1992, Latheef et al. 1993, Arthur 1999, Quaglia et al. 2001, Cao et al. 2005, and Takei et al. 2008). Although nanoparticle pesticides likely have already been used for pest control in the U.S., a thorough evaluation of their risk and exposure to humans has yet to be completed (Stone et al. 2010). Beyond human health impacts, risks to non-target organisms likely to come into contact with these compounds – including pollinators – have also not yet been thoroughly considered.  The possibility exists that nanoparticle pesticides or their derivatives may be toxic to and have adverse impacts on non-target pollinating insects that come into contact with them. Nanoparticle pesticides differ from conventional pesticides in size and surface characteristics, which may change their bioavailability or other properties (Stone et al. 2010). If nanoparticle pesticides accumulate in the environment, the impacts to a wide variety of non-target organisms could be significant. However, it is also possible that the impacts of these nanoparticle pesticides on pollinating insects will be less severe than the impacts of some conventional pesticides currently in use, because of their highly targeted delivery systems. | Arthur FH. 1999. Evaluation of an Encapsulated Formulation of Cyfluthrin to Control *Sitophilus oryzae* (L.) on Stored Wheat. *Journal of Stored Products Research*;35 159-166.  Cao Y, Huang L, Chen J, Liang J, Long S, Lu Y. 2005. Development of a Controlled Release Formulation Based on a Starch Matrix System. *International Journal of Pharmaceutics*;298 108–116.  Greene LC, Meyers PA, Springer JT, Banks PA. 1992. Biological Evaluation of Pesticides Released from Temperature-Responsive Microcapsules. *Journal of Agricultural and Food Chemistry*; 40 2274-2270.  Latheef MA, Dailey Jr OD, Franz E. 1993. Efficacy of Polymeric Controlled Release Formulations of Sulprofos Against Tobacco Budworm, *Heliothis virescens* (Lepidopetea:Noctuidae) on Cotton. In: Berger PD, Devisetty BN, Hall FR. (eds.) Pesticide Formulations and Applications Systems: 13^th^ volume, ASTM STP 1183. Philadelphia: American Society for Testing and Materials. p. 300-311.  National Nanotechnology Initiative. 2014. Available at: <https://www.whitehouse.gov/sites/default/files/microsites/ostp/NNI_FY15_Final.pdf>  Sekhon, BS. 2014. Nanotechnology in agri-food production: an overview. *Nanotechnology, Science and Applications*, 7, 31–53. http://doi.org/10.2147/NSA.S39406  Stone, DL, BJ Harper, I Lynch, K Dawson and SL Harper.  2010.  Exposure assessment: Recommendations for nanotechnology-based pesticides.  *International Journal of Occupational and Environmental Health* 16: 467-474.  Takei T, Yoshida M, Hatate Y, Shiomori K, Kiyoyama S. Preparation of Polylactide/Poly(Є-Caprolactone) Microspheres Enclosing Acetamiprid and Evaluation of Release Behavior. *Polymer Bulletin* 2008;61 391–397.  Vance, ME, Kuiken, T, Vejerano, EP, McGinnis, SP, Hochella, MF, Jr., Rejeski, D and Hull, MS. 2015. Nanotechnology in the real world: Redeveloping the nanomaterial consumer products inventory. *Beilstein Journal of Nanotechnology*, 6, 1769-1780.  Quaglia F, Barbato F, De Rosa G, Granata E, Miro A, La Rotonda MI. 2001. Reduction of the Environmental Impact of Pesticides: Waxy Microspheres Encapsulating the Insecticide Carbaryl. *Journal of Agricultural and Food Chemistry*;49 4808-4812. | |
| 5 | Nanoparticles | Nanoparticles are simply particles between 1 and 100 nanometers in size. There is much research into their biomedical, optical and electronic uses. Nanoparticles of silver have antibacterial properties, and are widely incorporated into pesticides that are applied to crops. However, they are not classified as active ingredients and thus are not subject to the usual regulatory process. Their persistence and environmental fate is almost entirely unknown. Their effects on pollinators have not been studied. One might suppose that they will be ingested by adult foraging bees or gathered in pollen and so contaminate larval food. Antibacterial properties may impact on bee gut flora. This is an area with huge knowledge gaps and poor regulation. | <http://modernfarmer.com/2015/01/everything-need-know-nanopesticides/> | |
| 6 | Below-ground effects on plant-pollinator interactions | Both soil nutrients and below-ground organisms, like nitrogen-fixing bacteria, arbuscular mycorrhizae and root-feeding herbivores, are known to influence floral traits, including the number and size of flowers, floral volatiles and attractants, and nectar and pollen quality (Lau & Stephenson 1994; Baude *et al.* 2011; Cardoza, Harris & Grozinger 2012; Barber & Soper Gorden 2014). These floral traits in turn influence pollinator visitation rates and behavior (Muñoz *et al.* 2005; Cardoza, Harris & Grozinger 2012) which can influence plant reproductive rates (Vázquez, Morris & Jordano 2005). Toxins in soils, including metals and pesticides, can also influence pollinators, either by bioaccumulating in nectar and pollen where they are picked up by foraging bees (Hladun *et al.* 2013; Chagnon *et al.* 2014), altering pollinator communities and visitation patterns (Moroń *et al.* 2012, 2014; Meindl & Ashman 2013, 2015), or altering the microbial composition of soils (Bünemann, Schwenke & Van Zwieten 2006; Lo 2010; Wyszkowska *et al.* 2012) which in turn affects pollinators (as above). Why below-ground effects on floral traits, pollinators and pollination is a horizon scan issue is because: (1) relatively little is known about this type of below- to above-ground connection, especially how soil conditions affect pollinator behavior, nutrition and health, which could in turn affect plant fitness and crop production(Barber & Soper Gorden 2014), and (2) many factors affect soil nutrients, soil organisms and soil toxins, including farming practices (Mader *et al.* 2002; Bünemann, Schwenke & Van Zwieten 2006; Hladun *et al.* 2013), agricultural inputs (Bünemann, Schwenke & Van Zwieten 2006; Lo 2010; Chagnon *et al.* 2014), urbanization or industrialization (Hladun, Parker & Trumble 2015) and climate change (Bellgard & Williams 2011); (3) these factors occur on large geographic scales which could translate into large impacts; and (4) alterations to soil characteristics could therefore affect key management issues, including crop pollination, pollination of rare or endangered plants, and reproduction and spread of invasive plants (Barber & Soper Gorden 2014).  **Additional notes**.  1. Effects of toxins on bee microbiome and health:  Metals like selenium, copper and cadmium can accumulate in pollen and nectar and be toxic to bees (Hladun *et al.* 2012; Hladun, Parker & Trumble 2015). Some microbes can grow *in vitro* in presence of these metals and may accumulate them. It is expected that these metals in the bee gut would alter the gut microbiome; based on preliminary data some of these microbes might create a protective function for bees by accumulating the toxins (Quinn McFrederick, pers. comm.), .), as has been found with related bacteria in the mouse gut (Zhai *et al.* 2014).  2. Effects of vermiculture on bee behavior and health  Vermiculture compost altered qualities of pollen and nectar that affected pollinator nutrition and behavior. Specifically, bumblebees feeding on VC amended cucumber flowers had larger and more active ovaries. Pollen from these plants had higher protein. Bumblebees increased visit length and reduced time to first discovery on VC plants.(Cardoza, Harris & Grozinger 2012)  3. While I focused on the above to below ground connection, of course soils are a critical substrate for ground-nesting bees, which comprise 70% of bee species. Relatively little is known about how physical characteristics of soil affect nesting of ground-nesting bees (Potts & Willmer 1997; Potts *et al.* 2005; Sardiñas & Kremen 2014), but bee-nesting success is likely to be influenced by land management factors that affect soil composition, moisture, compaction, slope and accessibility (e.g. Shuler, Roulston & Farris 2005). The physical characteristics of soils and how changes in them may affect bees could be added to this as part of the horizon scan issue (space permitting). | Barber, N. a. & Soper Gorden, N.L. (2014) How do belowground organisms influence plant-pollinator interactions? *Journal of Plant Ecology*, **8**, 1–11.  Baude, M., Leloup, J., Suchail, S., Allard, B., Benest, D., Mériguet, J., Nunan, N., Dajoz, I. & Raynaud, X. (2011) Litter inputs and plant interactions affect nectar sugar content. *Journal of Ecology*, **99**, 828–837.  Bellgard, S.E. & Williams, S.E. (2011) Response of mycorrhizal diversity to current climatic changes. *Diversity*, **3**, 8–90.  Bünemann, E.K., Schwenke, G.D. & Van Zwieten, L. (2006) Impact of agricultural inputs on soil organisms - A review. *Australian Journal of Soil Research*, **44**, 379–406.  Cardoza, Y.J., Harris, G.K. & Grozinger, C.M. (2012) Effects of Soil Quality Enhancement on Pollinator-Plant Interactions. *Psyche: A Journal of Entomology*, **2012**, 1–8.  Chagnon, M., Kreutzweiser, D., Mitchell, E. a. D., Morrissey, C. a., Noome, D. a. & Van der Sluijs, J.P. (2014) Risks of large-scale use of systemic insecticides to ecosystem functioning and services. *Environmental Science and Pollution Research*.  Hladun, K.R., Parker, D.R., Tran, K.D. & Trumble, J.T. (2013) Effects of selenium accumulation on phytotoxicity, herbivory, and pollination ecology in radish (*Raphanus sativus* L.). *Environmental Pollution*, **172**, 70–75.  Hladun, K.R., Parker, D.R. & Trumble, J.T. (2015) Cadmium, Copper, and Lead Accumulation and Bioconcentration in the Vegetative and Reproductive Organs of *Raphanus sativus*: Implications for Plant Performance and Pollination. *Journal of Chemical Ecology*, 386–395.  Hladun, K.R., Smith, B.H., Mustard, J. a., Morton, R.R. & Trumble, J.T. (2012) Selenium toxicity to honey bee (*Apis mellifera* l.) pollinators: Effects on behaviors and survival. *PLoS ONE*, **7**, 1–10.  Lau, T.-C. & Stephenson, A. (1994) Effects of soil phosphorus on pollen production, pollen size, pollen phosphorus content, and the ability to sire seeds in *Cucurbita pepo* (Cucurbitaceae). *Sexual Plant Reproduction*, **7**, 215–220.  Lo, C.-C. (2010) Effect of pesticides on soil microbial community. *Journal of environmental science and health. Part. B, Pesticides, food contaminants, and agricultural wastes*, **45**, 348–359.  Mader, P., Fliessbach, A., Dubois, D., Gunst, L., Fried, P. & Niggli, U. (2002) Soil fertility and biodiversity in organic farming. *Science*, **296**, 1694–1697.  Meindl, G.A. & Ashman, T.-L. (2013) The effects of aluminum and nickel in nectar on the foraging behavior of bumblebees. *Environmental pollution*, **177**, 78–81.  Meindl, G.A. & Ashman, T.-L. (2015) Effects of floral metal accumulation on floral visitor communities: introducing the elemental filter hypothesis. *American Journal of Botany*, **102**, 379–89.  Moroń, D., Grześ, I.M., Skórka, P., Szentgyörgyi, H., Laskowski, R., Potts, S.G. & Woyciechowski, M. (2012) Abundance and diversity of wild bees along gradients of heavy metal pollution. *Journal of Applied Ecology*, **49**, 118–125.  Moroń, D., Szentgyörgyi, H., Skórka, P., Potts, S.G. & Woyciechowski, M. (2014) Survival, reproduction and population growth of the bee pollinator, *Osmia rufa* (Hymenoptera: Megachilidae), along gradients of heavy metal pollution. *Insect Conservation and Diversity*, **7**, 113–121.  Muñoz, A. a., Celedon-Neghme, C., Cavieres, L. a. & Arroyo, M.T.K. (2005) Bottom-up effects of nutrient availability on flower production, pollinator visitation, and seed output in a high-Andean shrub. *Oecologia*, **143**, 126–135.  Potts, S.G., Vulliamy, B., Roberts, S., O’Toole, C., Dafni, A., Ne’Eman, G. & Willmer, P. (2005) Role of nesting resources in organising diverse bee communities in a Mediterranean landscape. *Ecological Entomology*, **30**, 78–85.  Potts, S.G. & Willmer, P. (1997) Abiotic and biotic factors influencing nest-site selection by *Halictus rubicundus*, a ground-nesting halictine bee. *Ecological Entomology*, **22**, 319–328.  Sardiñas, H. & Kremen, C. (2014) Evaluating nesting microhabitat for ground-nesting bees using emergence traps. *Basic and Applied Ecology*, **15**, 161–168.  Shuler, R.E., Roulston, T.H. & Farris, G.E. (2005) Farming practices influence wild pollinator populations on squash and pumpkin. *Journal of Economic Entomology*, **98**, 790–795.  Vázquez, D.P., Morris, W.F. & Jordano, P. (2005) Interaction frequency as a surrogate for the total effect of animal mutualists on plants. *Ecology Letters*, **8**, 1088–1094.  Wyszkowska, J., Kucharski, J., Kucharski, M. & Borowik, A. (2012) Effect of cadmium, copper and zinc on plants, soil microorganisms and soil enzymes. *Journal of Elemntology*, **18**, 769–796.  Zhai, Q., Wang, G., Zhao, J., Liu, X., Narbad, A., Chen, Y.Q., Zhang, H., Tian, F. & Chen, W. (2014) Protective effects of Lactobacillus plantarum CCFM8610 against chronic cadmium toxicity in mice indicate routes of protection besides intestinal sequestration. *Applied and Environmental Microbiology*, **80**, 4063–4071 | |
| 7 | Diffuse pollution: overlooked and underestimated? | Diffuse air pollution in the form of NOx and ozone can rapidly degrade airborne plant volatiles, both floral and vegetative in origin. Such effects occur selectively, affecting some volatile compounds more profoundly than others. These changes in the composition of floral volatile profiles can result in honey bees no longer being able to recognise that profile as a rewarding resource. How these effects impact real world foraging behaviour is not yet understood, however disruption of volatile chemical signalling has the potential to have profound effects on a wide range of pollinators and other insect groups. Furthermore, there is early evidence to demonstrate that these airborne pollutants can have direct effects on the on neurobiology and foraging success of honey bees and potentially other pollinating insects.  Given the global trend for increased diffuse pollution in most terrestrial ecosystems, coupled with a lack of understanding of direct and indirect impacts on pollinators highlights this as a key area needing concerted research. | Girling, R.D., Lusebrink, I., Farthing, E., Newman, T.A. & Poppy, G.M. (2013) Diesel exhaust rapidly degrades floral odours used by honeybees. *Scientific Reports* 3: 2779  Reitmayer, C., Newman, T. A., Girling, R., & Farthing, E. (2013). Diesel exhaust pollution affects learning abilities and leads to an altered stress response in the CNS of the honey bee (*Apis mellifera*). In GLIA (Vol. 61, pp. S72-S72). Hoboken, NJ USA: Wiley-Blackwell. | |
| 8 | Policy and market factors exacerbate simplification of agricultural landscapes | Globally, policy and market factors are increasingly leading to conversion of natural, semi-natural and diversified agricultural areas into highly simplified landscapes consisting of monocultures that are either devoid of floral resources for pollinators, or produce massive amounts of floral resources for short time periods (mass-flowering crops). These highly-simplified landscapes generally reduce the diversity and abundance of native pollinators (Kennedy *et al.* 2013), reduce pollination services from native pollinators to crops Klein *et al.* 2012; Garibaldi *et al.* 2014), and increase reliance on managed *Apis* pollinators (Kremen, Williams & Thorp 2002), which are suffering enhanced colony losses due to combined stressors of parasites and diseases, pesticides and land use change (Goulson *et al.* 2015). Although mass-flowering crops that characterize some monoculture landscapes may boost abundances of selected pollinator species (Herrmann *et al.* 2007; Holzschuh *et al.* 2013), they typically require use of an array of pesticides to control pests (Ref), and thus expose foraging pollinators to pesticides that impact populations of both managed and wild pollinators (Budge *et al.* 2015; Rundlöf *et al.* 2015). Further, increased abundances of wild pollinators from mass-flowering crops require continued existence of patches of semi-natural habitat (Diekotter *et al.* 2013; Kovács-Hostyánszki *et al.* 2013) and is likely only to benefit a small number of dominant generalist species (Kleijn *et al.* 2015).  This relatively well-known chain of ecological consequences resulting from landscape simplification is a horizon-scan issue because globalized social, political and economic factors are rapidly exacerbating the transformation of agricultural landscapes towards monocultures worldwide. Simultaneously, both grassroots initiatives and national and international mandates are pushing back against these forces to promote local and regional food systems that support sustainable, diversified agriculture.  Extensive consolidation in agri-food industries along the supply chain (i.e. companies that produce agricultural inputs of seeds, pesticides and fertilizers as well as companies that purchase, process and distribute food products) has led to unprecedented control over land access, land use and agricultural practices (Howard 2009; Oxfam 2013, http://www.worldwatch.org/node/5468). For example, three companies control about half of the global agrichemical market (<http://www.worldwatch.org/node/5468>) while 10 companies control a large portion of food processing, distribution and marketing (Fig 1). To gain advantage from economies of scale, agri-food industries favor large homogeneous production systems that produce large amounts of uniform products with relatively low transaction costs. These economic factors may have increased the consolidation of land-holdings that has occurred in many parts of the world (Macdonald, Korb & Hoppe 2013)(<http://ec.europa.eu/eurostat/statistics-explained/index.php/Agriculture_statistics_-_the_evolution_of_farm_holdings>), and associated landscape simplification as large land areas are farmed to a single commodity (e.g. corn and soy in large regions of the United States and Brazil; oil palm in large areas of Malaysia and Indonesia). Large transnational land deals for commodity crop and biofuels production now occupy over 24 million hectares, (<http://www.landmatrix.org/en/>), representing a new trend in large land-holdings and landscape simplification (e.g. 500,000 acres in Brazil for soybean to export to China; 540,000 acres in Liberia for rubber and palm oil, <http://www.wfs.org/futurist/january-february-2013-vol-47-no-1/food-fuel-and-global-land-grab>). Agri-food companies increasingly determine some or all practices on farmland, via contract farming (Prowse 2008), in which the buyer often provides the grower with seeds, seedlings or livestock, fertilizers, pesticides and technical assistance on credit, while assuring a given price for the crop product. Agri-food companies promote landscape simplification by encouraging the use of large areas of land for production of a single commodity, and/or by encouraging land management practices favoring the eradication of semi-natural habitat (Gennet *et al.* 2013). In turn, many governmental policies favor simplified landscapes producing a single commodity, such as subsidies and crop insurance programs in the United States for corn and soy commodities. | Budge, G.E., Garthwaite, D., Crowe, a., Boatman, N.D., Delaplane, K.S., Brown, M. a., Thygesen, H.H. & Pietravalle, S. (2015) Evidence for pollinator cost and farming benefits of neonicotinoid seed coatings on oilseed rape. *Scientific Reports*, 5, 12574.  Diekotter, T., Peter, F., Jauker, B., Wolters, V. & Jauker, F. (2013) Mass-flowering crops increase richness of cavity-nesting bees and wasps in modern agro-ecosystems. *GCB Bioenergy*, 219–226.  Garibaldi, L.A., Carvalheiro, L.G., Leonhardt, S.D., Aizen, M.A., Blaauw, B.R., Isaacs, R., Kuhlmann, M., Kleijn, D., Klein, A.M., Kremen, C., Morandin, L.A., Scheper, J. & Winfree, R. (2014) From research to action: enhancing crop yield through wild pollinators. *Frontiers in Ecology and the Environment*, 12, 439–447.  Gennet, S., Howard, J., Langholz, J., Andrews, K., Reynolds, M.D. & Morrison, S. (2013) Farm practices for food safety: an emerging threat to floodplain and riparian ecosystems. *Frontiers in Ecology and the Environment*, 11, 236–242.  Goulson, D., Nicholls, E., Botías, C. & Rotheray, E.L. (2015) Bee declines driven by combined stress from parasites , pesticides , and lack of flowers. *Science*, 347, -.  Herrmann, F., Westphal, C., Moritz, R.F.A. & Steffan-Dewenter, I. (2007) Genetic diversity and mass resources promote colony size and forager densities of a social bee (Bombus pascuorum) in agricultural landscapes. *Molecular Ecology*, 16, 1167–1178.  Holzschuh, A., Dormann, C., Tscharntke, T. & Steffan-Dewenter, I. (2013) Mass-flowering crops enhance wild bee abundance. *Oecologia*, 172, 477–484.  Howard, P.H. (2009) Visualizing Consolidation in the Global Seed Industry: 1996-2008. *Sustainability*, 1, 1266–1287.  Kennedy, C.M., Lonsdorf, E., Neel, M.C., Williams, N.M., Ricketts, T.H., Winfree, R., Bommarco, R., Brittain, C., Burley, A.L., Cariveau, D., Carvalheiro, L.G., Chacoff, N.P., Cunningham, S.A., Danforth, B.N., Dudenhöffer, J.-H., Elle, E., Gaines, H.R., Garibaldi, L.A., Gratton, C., Holzschuh, A., Javorek, S.K., Jha, S., Klein, A.M., Krewenka, K., Mandelik, Y., Mayfield, M.M., Morandin, L., Neame, L.A., Otieno, M., Park, M., Potts, S.G., Rundlöf, M., Saez, A., Steffan-Dewenter, I., Taki, H., Viana, B.F., Westphal, C., Wilson, J.K., Greenleaf, S.S., Taylor, H., Alana, L., Isaacs, R. & Kremen, C. (2013) A global quantitative synthesis of local and landscape effects on wild bee pollinators in agroecosystems. *Ecology Letters*, 16, 584–599.  Kleijn, D., Winfree, R., Bartomeus, I., Carvalheiro, L.G., Henry, M., Isaacs, R., Klein, A.-M., Kremen, C., M’Gonigle, L.K., Rader, R., Ricketts, T.H., Williams, N.M., Lee Adamson, N., Ascher, J.S., Báldi, A., Batáry, P., Benjamin, F., Biesmeijer, J.C., Blitzer, E.J., Bommarco, R., Brand, M.R., Bretagnolle, V., Button, L., Cariveau, D.P., Chifflet, R., Colville, J.F., Danforth, B.N., Elle, E., Garratt, M.P.D., Herzog, F., Holzschuh, A., Howlett, B.G., Jauker, F., Jha, S., Knop, E., Krewenka, K.M., Le Féon, V., Mandelik, Y., May, E. a, Park, M.G., Pisanty, G., Reemer, M., Riedinger, V., Rollin, O., Rundlöf, M., Sardiñas, H.S., Scheper, J., Sciligo, A.R., Smith, H.G., Steffan-Dewenter, I., Thorp, R., Tscharntke, T., Verhulst, J., Viana, B.F., Vaissière, B.E., Veldtman, R., Westphal, C. & Potts, S.G. (2015) Delivery of crop pollination services is an insufficient argument for wild pollinator conservation. *Nature Communications*, 6, 7414.  Klein, A.-M., Brittain, C., Hendrix, S.D., Thorp, R., Williams, N. & Kremen, C. (2012) Wild pollination services to California almond rely on semi-natural habitat. *Journal of Applied Ecology*, 49, 723–732.  Kovács-Hostyánszki, A., Haenke, S., Batáry, P., Jauker, B., Báldi, A., Tscharntke, T. & Holzschuh, A. (2013) Contrasting effects of mass-flowering crops on bee pollination of hedge plants at different spatial and temporal scales. *Ecological Applications*, 23, 1938–1946.  Kremen, C., Williams, N.M. & Thorp, R.W. (2002) Crop pollination from native bees at risk from agricultural intensification. *Proceedings of the National Academy of Sciences*, 99, 16812–16816.  Macdonald, J.M., Korb, P. & Hoppe, R.A. (2013) Farm Size and the Organization of U.S. Crop Farming. *Economic Research Report*, 1–61.  Oxfam. (2013) *Behind the Brands*.  Prowse, M. (2008) *Contract Farming in Developing Countries - A Review*. Paris, France.  Rundlöf, M., Andersson, G.K.S., Bommarco, R., Fries, I., Hederström, V., Herbertsson, L., Jonsson, O., Klatt, B.K., Pedersen, T.R., Yourstone, J. & Smith, H.G. (2015) Seed coating with a neonicotinoid insecticide negatively affects wild bees. *Nature*. | |
| 9 | Soybean crop expansion worldwide | Agriculture growth has been pointed out as a threat to pollinators due to deforestation, soil disturbance and pesticide use among other practices (Freitas 2009). Soybean, for example, has shown 36% increase in production during the decade 2000-2009, but 81% of this figure was due to expansion of cropped land while yield increased only 6% contributing only 19 % to the world soybean production increment (Masuda and Goldsmith 2009). Also, soybean decreases significantly precipitation after an extension in cultivated area due the very high albedo of this crop (Costa et al. 2007, Barona et al 2010). Milfont et al (2013) demonstrated that honey bees and wild bees can potentially increase soybean yield to an extra value of US$ US$ 17.461 billion to the world economy, equivalent to expand the soybean area in approximately 18.7 million ha. But major soybean growers are expanding their crops overseas; China is now cultivating soybean in Laos, Cambodia, Africa, South America, Central Asia and Ukraine (Yale Global 2014). But the pollinator unfriendly environment of soybean crops is believed to harm most potential pollinators and the impact of the continuous growth of soybean cropped areas and enlargement of fields on local pollinators have been neglected and are unknown. | Barona E, Ramankutty N, Hyman G, Coomes OT (2010) The role of pasture and soybean in deforestation of the Brazilian Amazon. *Environ. Res. Lett*. 5 024002 (9pp). doi:10.1088/1748-9326/5/2/024002  Costa MH, Yanagi SNM, Souza PJOP, Ribeiro A, Rocha EJP (2007) Climate change in Amazonia caused by soybean cropland expansion, as compared to caused by pastureland expansion. *Geophysical Research Letters* 34, L07706, doi:10.1029/2007GL029271  Freitas BM, Imperatriz-Fonseca VL, Medina LM, Kleinert AMP, Galetto L, Nates-Parra G, Quezada-Euán JJG (2009) Diversity, threats and conservation of native bees in the Neotropics. *Apidologie* 40:332–346.  Masuda T, Goldsmith PD (2009) World soybean production: area harvested, yield, and long-term projections. *Int Food Agribus Manag Rev* 12:143–162.  Milfont M de O, Rocha EEM, Lima AON, Freitas (2013) Higher soybean production using honeybee and wild pollinators, a sustainable alternative to pesticides and autopollination. *Environ Chem Lett* 11:335–334. doi: 10.1007/s10311-013-0412-8  Yale Global (2014) Chinese Agriculture Goes Global. <http://yaleglobal.yale.edu/content/chinese-agriculture-goes-global>. Acessed 25 August 2015. | |
| 10 | Reduction or even removal of glyphosate* | The most widely used herbicide, glyphosate, has recently been classed as probably carcinogenic (Guyton et al. 2015). The research was conducted by the International Agency for Research on Cancer (IARC), which is the cancer-research arm of the World Health Organisation. Their finding adds to other recent studies of the side effects of this widely used herbicide, which have also revealed that it has impacts on below ground interactions between earthworms and mycorrhizal fungi (Zaller et al. 2014). One possible consequence of such findings (especially the probable carcinogenic effects) is a reduction in use of glyphospate in the USA and other major food-producing regions around the world. It could also reduce the productivity of crops genetically engineered for glyphosate resistance. This could generate either positive or negative consequences for pollinators. Herbicide use has large indirect effects on pollinators in agricultural landscapes, through loss of flowers. | Guyton et al. 2015. *Lancet Oncol*. http://dx.doi.org/10.1016/S1470-2045(15)70134-8  Zaller et al. 2014. Glyphosate herbicide affects belowground interactions between earthworms and symbiotic mycorrhizal fungi in a model ecosystem. *Scientific Reports* 4: 5364. doi:10.1038/srep05634 | |
| 11 | Potential loss of floral resources for pollinators within and adjacent to agricultural lands through adoption of forthcoming ‘next generation’ genetically engineered crops and associated herbicide use | The U.S. Department of Agriculture has recently approved a suite of ‘next generation’ genetically engineered (GE) herbicide resistant corn and soybeans [developed by Dow Agrosciences](http://www.regulations.gov/#!docketBrowser;rpp=25;so=DESC;sb=postedDate;po=0;dct=SR;D=APHIS-2013-0042) and soy and cotton [developed by Monsanto](http://www.aphis.usda.gov/brs/fedregister/BRS_20150120a.pdf), which will be sold in conjunction with new combinations of herbicides. These GE crops are resistant to the herbicides 2,4-D and glyphosate (Enlist^TM^ by Dow Agrosciences) and dicamba and glyphosate (Roundup Ready Xtend^TM^ by Monsanto).  The use of herbicides is expected to increase with the adoption of these ‘next generation’ GE crops (Mortensen et al. 2012). Dicamba and 2,4-D are already among the leading herbicides that cause drift-related crop injury because of their volatility (Freese and Crouch 2015 pp. 67-71 and references therein). Because of the increased volatility of dicamba and 2,4-D over glyphosate (which is currently the most widely used herbicide in the U.S.), the loss of flowering weeds and wildflowers growing within and adjacent to agricultural land is expected to be more significant than at present. Expanded use of 2,4-D and dicamba could have a major negative impact on populations of insect pollinators collecting nectar and pollen from weeds and wildflowers growing near crops.  Globally, more than 170 million hectares are planted in GE crops (Marshall 2013), and the U.S. has more than 70 million hectares planted in GE crops (James 2013), with the vast majority in soy, corn, and cotton. Introduced in the 1990s, now approximately 90% of all soy, corn, and cotton in the U.S. is genetically engineered to resist the herbicide glyphosate (USDA 2015). With the widescale adoption of GE crops, the use of glyphosate on corn and soy in the U.S. has increased from 10 million pounds in 1995 to 200 million pounds in 2013 (Center for Biological Diversity et al. 2014 and references therein, p. 48). Though the loss of most species of flowering plants within and around corn and soy fields due to increased use of glyphosate has not been quantified, researchers have documented a 64% loss of milkweed from the Midwestern U.S. landscape from 1999-2012 (Pleasants 2015). Common milkweed is the primary host plant for monarch butterflies, and a significant decline in the monarch population since 1994 has been linked to the use of this weed control technology (Pleasants 2015, Pleasants and Oberhauser 2012). Though the monarch butterfly’s role as a pollinator is largely unknown, one can imagine that the non-target effects of increasingly efficient weed control technology may have a dramatic impact on pollinator populations within the U.S. landscape, as well as in other regions where GE crops are grown. | Center for Biological Diversity, Center for Food Safety, Xe­rces Society for Invertebrate Conservation, and Dr. L. Brower. 2014. “Petition to protect the monarch butter­fly (*Danaus plexippus plexippus*) under the Endangered Species Act.” Report submitted to the United States Sec­retary of the Interior, Washington, D.C., 26 August 2014. 159 pp. Available at http://www.xerces.org/wp-content/ uploads/2014/08/monarch-esa-petition.pdf  Freese, B. and M. Crouch. 2015. Monarchs in peril: Herbicide-resistant crops and the decline of monarch butterflies in North America. Center for Food Safety, February 2015. Available at: <http://www.centerforfoodsafety.org/files/cfs-monarch-report_4-2-15_design_87904.pdf>  James, Clive. 2013. Global Status of Commercialized Biotech/GM Crops: 2013. ISAAA Brief  No. 46. ISAAA: Ithaca, NY. Available at: <http://www.isaaa.org/resources/publications/briefs/46/executivesummary/>  Marshall, 2013. *Nature Biotechnology* 31, 278 doi:10.1038/nbt.2552 <http://www.nature.com/nbt/journal/v31/n4/box/nbt.2552_BX4.html>  Mortensen D.A., J.F. Egan, B.D. Maxwell, M.R. Ryan, and R.G. Smith. 2012. Navigating a Critical Juncture for Sustainable Weed Management. *BioScience* 62: 75-84.  Pleasants, J.M. 2015. Monarch Butterflies in Agriculture. In: Oberhauser K.S., K.R. Nail, and S. Altizer, editors. Monarchs in a Changing World: Biology and Conservation of an Iconic Butterfly. New York (NY): Cornell University Press. pp. 169-178.  Pleasants, J.M., and K.S. Oberhauser. 2012. Milkweed loss in agricultural fields because of herbicide use: effect on the monarch butterfly population. *Insect Conservation and Diversity* 6:135–144. Available from http://doi.wiley.com/10.1111/j.1752-4598.2012.00196.x  US Department of Agriculture. 2015. Adoption of genetically engineered crops in the U.S.: Recent trends in GE Adoption. Available at: http://www.ers.usda.gov/data-products/adoption-of-genetically-engineered-crops-in-the-us/recent-trends-in-ge-adoption.aspx#.U8xmYvldVJA | |
| 12 | Agricultural policy leading to intensification/abandonment/reforestation | Global agriculture is expanding and intensifying to meet food and bioenergy demands. Policy decisions can influence land-use patterns (Tilman et al. 2011) which can influence both resource availability for pollinators, and their exposure to potentially harmful pesticides. For example, changes to the EU Common Agricultural Policy (CAP) may result in the abandonment of pastoral land at a European scale (Renwick et al. 2013), whilst changes in policy at national scales or outside the EU may have the opposite effect. Abandonment can lead to scrub formation, which may be beneficial for some pollinators, depending on scrub type (Potts et al. 2006, Fuentes-Montemayor et al. 2010). Intensification often leads to decline in biodiversity, including pollinators and insect-pollinated plants (Clough et al. 2014). In Ireland, for example, there are plants to increase and intensify dairy production (DAFM 2010). Pastures offer relatively stable environments for pollinators due to the lack of annual tillage (Potts et al. 2009, Power and Stout 2011), but intensive fertiliser and herbicide application, as well as high stocking densities can reduce the availability of resources (flowers, nesting sites for Hymenoptera and larval resources for Lepidoptera and Diptera). Reforestation with conifers can provide little in terms of resources for pollinators, whilst reforestation of native mixed woodlands may provide benefits. | Clough, Y., Ekroos, J., Báldi, A., Batáry, P., Bommarco, R., Gross, N., Holzschuh, A., Hopfenmüller, S., Knop, E., Kuussaari, M., Lindborg, R., Marini, L., Öckinger, E., Potts, S.G., Pöyry, J., Roberts, S.P.M., Steffan-Dewenter, I., Smith, H.G., 2014. Density of insect-pollinated grassland plants decreases with increasing surrounding land-use intensity. *Ecology Letters* 17, 1168-1177.  DAFM, 2010. Food Harvest 2020 - A vision for Irish agri-food and fishery. Department for Agriculture. Food and the Marine.  Fuentes-Montemayor, E., Goulson, D., Park, K.J., 2010. The effectiveness of agri-environment schemes for the conservation of farmland moths: assessing the importance of a landscape-scale management approach. *Journal of Applied Ecology*,  Potts, S.G., Petanidou, T., Roberts, S., O’Toole, C., Hulbert, A., Willmer, P., 2006. Plant-pollinator biodiversity and pollination services in a complex Mediterranean landscape. *Biological Conservation* 129, 519-529.  Renwick, A., Jansson, T., Verburg, P.H., Revoredo-Giha, C., Britz, W., Gocht, A., McCracken, D., 2013. Policy reform and agricultural land abandonment in the EU. *Land Use Policy* 30, 446-457.  Potts, S.G., Woodcock, B.A., Roberts, S.P.M., Tscheulin, T., Pilgrim, E.S., Brown, V.K., Tallowin, J.R., 2009. Enhancing pollinator biodiversity in intensive grasslands. *Journal of Applied Ecology* 46, 369-379.  Power, E.F., Stout, J.C., 2011. Organic dairy farming: impacts on insect–flower interaction networks and pollination. *Journal of Applied Ecology* 48, 561–569.  Tilman, D., Balzer, C., Hill, J., Befort, B.L., 2011. Global food demand and the sustainable intensification of agriculture. *Proceedings of the National Academy of Sciences* 108, 20260-20264 | |
| 13 | Land sparing (setting aside land for biodiversity conservation and intensifying production on remaining land) | Maintaining functional diversity of pollinators requires mosaics of land use at landscape scale (Forrest et al. 2015). Agricultural and nature conservation policies do not necessarily align to provide the best landscape composition and configuration for pollinators (Hodge et al. 2015). Land sparing (Phalan et al. 2011) may mean there are few resources for pollinators in intensified agricultural/forestry areas (which may have implications for production of animal-pollinated crops), and areas set aside for biodiversity conservation may not be big or diverse enough to support diverse pollinator communities. The scale at which pollinator communities respond to land use is not well understood, and may not be the same for differing functional groups (Steckel et al. 2014). Thus the scale at which land sparing is implemented is important. | Forrest, J.R.K., Thorp, R.W., Kremen, C., Williams, N.M., 2015. Contrasting patterns in species and functional-trait diversity of bees in an agricultural landscape. *Journal of Applied Ecology* 52, 706-715  Hodge, I., Hauck, J., Bonn, A., 2015. The alignment of agricultural and nature conservation policies in the European Union. *Conservation Biology* 29, 996-1005  Phalan, B., Onial, M., Balmford, A., Green, R.E., 2011. Reconciling food production and biodiversity conservation: land sharing and land sparing compared. *Science* 333, 1289-1291  Steckel, J., Westphal, C., Peters, M.K., Bellach, M., Rothenwoehrer, C., Erasmi, S., Scherber, C., Tscharntke, T., Steffan-Dewenter, I., 2014. Landscape composition and configuration differently affect trap-nesting bees, wasps and their antagonists. *Biological Conservation* 172, 56-64 | |
| 14 | Lack of investment in research into sustainable farming methods | Governments indicate that they support moves to reduce pesticide use; in the EU, all farms are legally bound to use “Integrated Pest Management”, a term originally used to describe approaches to farming which used pesticides only as a last resort. The UK’s National Pollinator Strategy argues that we should move towards reduced pesticide use, as does the USA’s new pollinator action plan, yet agricultural policy favours moves towards larger fields and larger holdings, often owned or run by international agricultural businesses focussed on profitability rather than sustainability. Most investment in agronomic research comes from industry, and tends to be focussed on squeezing ever-higher yields through high inputs. There are few avenues of funding for independent research into the more sustainable approaches the governments say they support. Industry is unlikely to invest in ways to reduce inputs, for supply of those inputs provides much of its profit. Unless governments are willing to invest in research and support for sustainable farming systems with reduced inputs, systems that conserve the soil, and minimise impacts upon wildlife such as bees, then farming will continue to move further towards high-input, intensive approaches. |  | |
| 15 | Risks and opportunities of cutting pollinators out of food production | It is well established that the majority of global food crops are dependent, to varying degrees, on biotic pollination. Given increasing concerns about declines in pollinators and increased demand for crop production, could the reliance on pollinators as an agricultural input be reduced or even cut out? Plant breeding technology, both conventional and fast emerging new molecular approaches, could provide varieties with reduced reliance on biotic pollination by switching to self or wind-pollination. A report by STEP identified that for industry “*breeding for varieties that are less dependent on insect pollination is still a goal for several entomophilous crops*”, and progress has been made in this direction for some varieties of almond, sweet cherry and apricot.  Potential benefits to growers and consumers could be reduced yield variability and reduced risks to pollination deficits in future, but what would be the trade-off in yield and quality? What are the risks and barriers associated with breeders reducing the reliance on pollinators (e.g. technological, market, environmental), and conversely what are the potential benefits (e.g. increased stability, more profit, poverty alleviation)? Basic empirical data is lacking on all these aspects, and with increasing pressure on farming to produce more food, coupled with accelerating innovations in plant breeding, a fundamental switch in the role of pollinators in global agriculture may happen before we fully understand the risks and opportunities. | Audergon JM, Duffillol JM, Pinet C, Blanc P. 1999. Pollination of three main apricot cultivars in France. *Acta Hort*. 488 :327-333.  Ortega E, Egea J, Cánovas JA, Dicenta F. 2002. Pollen tube dynamics following half- and fully-compatible pollinations in self-compatible almond cultivars. *Sex. Plant Reprod*. 15 :47-51.  Sonneveld T, Tobutt KR, Vaughan SP, Robbins TP. 2005. Loss of pollen-S function in two self-compatible selections of *Prunus avium* is associated with deletion/mutation of an S haplotype-specific F-box gene. *Plant Cell* 17 :37-51.  Vaissière B. et al. (2011) Analysis of the effectiveness of measures mitigating for pollination loss in crops and wild plants. STEP Deliverable 4.4 | |
| 15 | Possible horticultural industry responses to pollinator limitation: pollination free crops | Developing new cultivars that do not need insect pollination for fruit and seed set could result in new planting crop varieties for which investment in floral structures has been actively selected out, or in which floral traits have drifted or been indirectly selected to no-longer benefit pollinators. This kind of process has occurred in the development of many horticultural garden flower varieties. Genomic doubling can make larger flowers and fruits, but the fruits are inaccessible to pollinators, or produce nectars of no energetic value. Widespread deployment of crops that did not need insect pollination is a likely reaction by the horticultural industry to pollinator limitation, but it will reduce the imperative to conserve wild pollinators and it could also make habitats even more inhospitable to natural pollinators. | Yao J.-L., Dong Y-H & Morris B.A.M 2001 Parthenocarpic apple fruit production conferred by transposon insertion mutations in a MADS-box transcription factor. *PNAS* 98 1306-1311 | |
| 16 | Precision agriculture could improve pollination & reduce harm to pollinators | With rising labour costs and limits on productive land area, horticultural and agricultural industries are investing in the development of new automated farming technologies (Calvin & Martin 2010; Sarig et al 2010; Zhang et al 2002). Artificial pollination techniques – including air-blown, liquid and physical application methods - are already utilised in a number of crops, and could be further automated to increase the precision of pollen application (Craig et al 1988; Razeto et al 2005; Kuru 1995). However, seed set data from assessments of artificial pollination demonstrate that the presence of honey bees improves pollination due to the collection of excess pollen and directed flower-visiting activity of honey bees (R.M. Goodwin, pers. comm. 2015). It is likely that honey bees and other highly-evolved insect pollinators will remain important to crop production by complementing artificial pollination, even in a largely automated future. Developments in automation of farm systems also provide opportunities to re-design farming to reduce harmful environmental effects and thus benefit diverse pollinator species. Automated detection and targeted control of pest species could largely eliminate non-target deaths from pesticides, and precise GPS-programmed control systems could avoid damage to pollinator habitat from large farm vehicles in situations where accidental damage from human error can be a problem (Acaccia et al 2003). Many crops are being increasingly grown under protective canopies, which will require a reconsideration of the best pollination strategies (Wittwer & Castilla 1995); including the use of artificial pollination methods and the use of new pollinator species more willing to pollinate under covered canopies than the European honey bee. | Calvin L., Martin P. (2010) *The U.S. Produce Industry and Labor: Facing the Future in a Global Economy.* Economic Research Report No. ERR-106. US Department of Agriculture, Economic Research Services: 2010;57p. <http://www.ers.usda.gov/media/135123/err106.pdf>.  Sarig Y., Thompson J.F., Brown G.K. (2010) *Alternatives to Immigrant Labor? The Status of Fruit and Vegetable Harvest Mechanization in the United States.* Centre for Immigration studies: Washington DC, December 2010; <http://www.cis.org/FarmMechanization-ImmigrationAlternative>  Zhang N., Wang M., Wang N. (2002) Precision agriculture – a worldwide overview. *Computers and Electronics in Agriculture*, 36:113-132.  Craig J.L., Stewart A.M., Pomeroy N., Heath A.C.G., Goodwin R.M. (1988) A review of kiwifruit pollination: Where to next? *New Zealand Journal of Experimental Agriculture* 16:385-399, DOI: 10.1080/03015521.1988.10425667  Razeto B., Reginato G., Larraín A. (2005) Hand and Machine Pollination of Kiwifruit, *International Journal of Fruit Science* 5:37-44, DOI: 10.1300/J492v05n02_05  Kuru C. (1995) Artificial pollination of pistachio trees under unsufficient pollination conditions. *Acta Horticulturae*. 419:121-124 DOI: 10.17660/ActaHortic.1995.419.18  Acaccia G.M, Michelini R.C., Molfino R.M., Razzoli R.P. (2003) Mobile robots in greenhouse cultivation: inspection and treatment of plants. Proccedings of ASER 2003, 1^st^ International Workshop on Advances in Services in Robotics, 13-15 March 2003, Bardolino, Italy.  Wittwer S.H., Castilla N. (1995) Protected Cultivation of Horticultural Crops Worldwide. *HortTechnology* 5:6-23 | |
| 17 | Corporate farming could see effective alternative pollination systems adopted rapidly | In some regions and crop industries, there is an ongoing shift from owner-operated, small-scale farms to corporate ownership of networks of orchards and farms (Deininger & Byerlee 2012; UNCTAD 2009), with an accompanied push for optimised, scale-able tools and solutions. These corporate entities want to know which single pollination method is most efficient, so that it can be applied on a large scale. If the economic case can be made for diverse pollinators (demonstrating quantifiable benefits given the cost of implementing and monitoring the systems), then a corporate farmer is likely to be more willing than traditional farmers to rapidly adopt this alternative approach to pollination. Without this clear economic case, pollination by high densities of managed honey bee hives will continue to dominate large-scale cropping. | Deininger K., Byerlee D. (2012) The Rise of Large Farms in Land Adundant Countries: Do They Have a Future? *World Development*, 40:701-714.  UNCTAD (2009) *World Investment report 2009*: *Transnational corporations, agricultural production, and development.* United Nations, New York & Geneva. | |
| 18 | New positions open for alternative pollinators: must have good credentials | Global demand for pollination services is on the rise as greater areas of pollinator-dependent crops are grown each year, yet supplies of managed and wild pollinators are in decline in some regions of the world. Relatively few species of pollinators are currently managed (eastern and western honeybees, and a few species of bumblebees, stingless bees and solitary bees), while the pool of wild species which could potentially be developed for management is huge. There are a number of well documented challenges associated with the currently available cohort of managed pollinators including: insufficient availability (in terms of numbers, location and time in season), risks of disease transmission within managed and across to wild populations, gene flow between managed and wild conspecifics, invasions and competition with wild fauna, and that some managed species inefficient pollinators of target crops.  Developing new species of pollinators could help meet local demands, and may provide species which are better matched to pollinating target crops (e.g. through behavioural or phenological traits). However, the same risks outlined above for current managed species would also apply here. Several candidate species have been identified and are currently being assessed for feasibility for local and/or industrial production, and research is needed to both help direct the development and commercialisation, and also to ensure associated risks (both environmental and economic) are well understood before new managed species are rolled out to help improve crop production. | Breeze T.D. et al. (2014) Agricultural policies exacerbate pollination service supply-demand mismatches across Europe. *PLoS ONE* 9(1): e82996  Delaplane K.S. & Mayer D.F. (2000) *Crop pollination by bees*. CABI Publishing, New York, USA. 332  Fuerst M.A. et al. (2014) Disease associations between honeybees and bumblebees as a threat to wild pollinators. *Nature* 506, 364-366  Velthuis H.H.W & Doorn A. van. (2006) A century of advances in bumblebee domestication and the economic and environmental aspects of its commercialization for pollination. *Apidologie* 37 :421-451. | |
| 19 | Possible horticultural industry responses to pollinator limitation: bees in boxes | Bumble bees in shoe boxes. Shoe bees are great for glasshouse tomatoes, but ultimately not good at all for an argument that we need to maintain natural numerous and diverse bumble bee populations. We are also seeing production of solitary bee species for purchase. Big Ag is also actively researching methods for mass production of honey bees using industrial methods and on industrial scale as a solution to the current pollination bottle-neck for almond. Should this battery bee initiative succeed we would see for the first time a truly domesticated honey bee, which would have negative consequences for any argument to maintain wild honey bee populations, and possible negative consequences for the genetic strength of wild bee populations as a result of introgression with domesticated genotypes. | Heard T. & Dollin A. 2013 Crop pollination with Australian stingless bees. The Native Bees of Australia Booklet 6 | |
| 20 | GMO honey bees: a boon to pollination | Selective breeding of the honey bee (*Apis mellifera*) to withstand pathogens and pesticides is underway (EU project Smart Bee: <http://www.smartbees-fp7.eu>; Canadian project Bee Doctor: <http://zayedlab.apps01.yorku.ca/wordpress/%3Fpage_id=8>). Recombinant honey bees with beneficial loci at important QTL loci for ‘health’ may become available (Grozinger & Robinson 2015). If beneficial alleles can be driven into local populations of honey bees, either through their selective advantage or artificial means, then the honey bee population of a region could be enhanced and, thereby, the ecosystem service of pollination. | Grozinger CM, Robinson GE (2015) The power and promise of applying genomics to honey bee health. *Current Opinion in Insect Science* in press | |
| 21 | Natural selection and apiculture: breeding | Since royal subfamilies are rare in honey bee colonies (Moritz et al 2005), human choice of larvae for queen rearing based on appropriate age alone is likely to miss those and instead offers only suboptimal choices for the bees. Moreover, breeding for *V. destructor*-resistance over >20 years has still not resulted in survival of untreated colonies, but natural selection has delivered (Rosenkranz et al. 2010), thereby suggesting that breeders should choose traits favored by natural selection. This suggests fundamental conceptual flaws in commercial honey bee queen rearing and breeding. Moreover, natural biodiversity of honey bees is severely threatened because breeding mainly focusses on two subspecies *A. m. carnica* and *A. m. ligustica* although recent data suggest considerable local adaptations (Büchler et al. 2014). | Büchler, R. et al. (2014) The influence of genetic origin and its interaction with environmental effects on the survival of *Apis mellifera* L. colonies in Europe *J. Apic. Res*. 53(2): 205-214  Moritz, R.F.A. et al. (2005) Rare royal families in honeybees, *Apis mellifera*. *Naturwissenschaften* 92: 488-491  Rosenkranz P, et al. (2010) The biology and control of *Varroa destructor*. *J. Invertebr. Pathol*. 103, 96-119 | |
| 22 | Entomovectoring | A relatively well known and carefully developed technology, but only well known within the pollinator research community. The approach has been demonstrated in flowering crops in greenhouses and recently field trials (see refs). A company, Bee Vector Technology (http://modernfarmer.com/2014/07/putting-bees-back-work/), has carried out 7 years of testing and claimed to see improved efficiency of pesticide application and increased yields. Given recent proof of efficacy, this is likely to become as widespread as managed pollination for some, if not all crops they are used for. Have we fully examined the risks of this taking place on a large scale, including in the field. What will it do to natural microbial communities in flowers? The control agents are very likely to be transferred to the wild plant community, surely. | Shipp L; Kapongo JP; Park H-H; Kevan P (2012) Effect of bee-vectored *Beauveria bassiana* on greenhouse beneficials under greenhouse cage conditions; *Biological Control*; 63:135-142  Mommaerts V, Put K, Smagghe G (2011) *Bombus terrestris* as pollinator-and-vector to suppress Botrytis cinerea in greenhouse strawberry, *Pest Management Science*, 67:1069-1075  Mommaerts V, Put K, Smagghe G (2011) Entomovectoring in plant protection.  Reeh KW; Hillier NK; Cutler GC (2014) Potential of bumble bees as bio-vectors of *Clonostachys rosea* for Botrytis blight management in lowbush blueberry; *Journal of Pest Science*; 87:543-550 | |
| 23 | Reduced budgets for public greenspace management | Public greenspace, including parks and other amenity grassland, makes up a large proportion of urban areas (Baldock *et al.* in prep). The responsibility for managing these areas often lies with local authorities and management can be an important factor in the biodiversity of greenspace sites (Schwartz *et al.* 2013). However, at least in the UK, local authorities are under pressure to save costs with large reductions in greenspace management budgets due to decreased funding from central government. Reduced amenity grassland management could have both positive and negative impacts for pollinators. Less frequent mowing could potentially benefit pollinators in the short term. Garbuzov *et al.* (2015) found that reduced mowing regimes led to greater flower abundance and numbers of flower-visiting insects in a suburban park. However, mowing may help to reduce competition from grasses thus infrequent mowing could lead to longer term declines in floral abundance (Williams *et al.* 2007). Other consequences of decreases in greenspace management budgets include reduced litter collection and infrastructure maintenance which are likely to lead to increased vandalism and vermin levels (T. Ahjokoski, pers. comm.). Furthermore budgets will be reduced for ornamental plantings, which are an opportunity for incorporating pollinator forage plants into public greenspace. These factors have the potential to lead to amenity grassland sites becoming increasingly inhospitable, both for pollinators and for people (T. Ahjokoski, pers. comm.). | Garbuzov et al. (2015) Public approval plus more wildlife: twin benefits of reduced mowing of amenity grass in a suburban public park in Saltdean, UK. *Insect Conservation and Diversity* **8**: 107-119.  Schwartz A, Muratet A, Simon L & Julliard R. (2013) Local and management variables outweigh landscape effects in enhancing the diversity of different taxa in a big metropolis. *Biological Conservation* **157**: 285-292.  Williams DW, Jackson LL & Smith DD (2007) Effects of frequent mowing on survival and persistence of forbs seeded into a species-poor grassland. *Restoration Ecology* **15**(1) 24-33 | |
| 24 | Green roofs as potential pollinator habitat | Green roofs on buildings have been found to have both economic and environmental benefits including increased roof longevity, reduced stormwater runoff and decreased building energy consumption (Clark *et al.* 2008). A green roof is a roof of a building that is partially or completely covered with vegetation and a growing medium. Converting rooftops into green roofs has the potential to benefit pollinators and other invertebrates, turning manmade structures into ecological habitats. Green roofs are utilised by a range of invertebrate taxa (Braaker *et al.* 2014) and those containing diverse floral mixes can attract a range of native bee species (Colla *et al.* 2009, Tonietto *et al.* 2011). Although some studies have found lower numbers and diversity of bees on green roofs compared to ground level, the insects present can provide sufficient pollinator services for plants, including native species (Ksiazek *et al.* 2012). Therefore green roofs have the potential to increase pollinator habitat, particularly in densely built environments such as towns and cities. Legislation in some regions is helping to promote green roof creation. For example green roofs are mandatory on new buildings with flat roofs in Basel, Switzerland (Brenneisen *et al.* 2006) and a French law introduced in 2015 mandates that new buildings in commercial zones must be partially covered in either plants or solar panels (Agence France-Presse 2015). | Agence France-Presse (2015) France decrees new rooftops must be covered in plants or solar panels. The Guardian. Accessed 9^th^ September 2015 url: http://www.theguardian.com/world/2015/mar/20/france-decrees-new-rooftops-must-be-covered-in-plants-or-solar-panels  Brenneisen S (2006) Space for urban wildlife: Designing green roofs as habitats in Switzerland. *Urban Habitats*: **4**, 27–36.  Clark C, Adriaens P & Talbot B (2008) Green roof valuation: a probabilistic economic analysis of environmental benefits. *Environmental Science and Technology* **42**: 2155-2161.  Colla, S. R., Willis, E., & Packer, L. (2009). Can green roofs provide habitat for urban bees (Hymenoptera: Apidae)? *Cities and the Environment*: **2**(1) 4.  Braaker S, Ghazoul J, Obrist MK & Moretti M (2014) Habitat connectivity shapes urban arthropod communities: the key role of green roofs. *Ecology* **95**(4): 1010-1021.  Tonietto R, Fant J, Ascher J, Ellis K & Larkin D (2011) A comparison of bee communities of Chicago green roofs, parks and prairies. *Landscape and Urban Planning* **103**(1): 102-108 | |
| 25 | Climate change causing changes in crop distribution, leading to changes in managed pollinator distributions | In South Africa, this is a particular problem, because the two subspecies *Apis mellifera capensis* and *Apis mellifera scutellata* are both indigenous but occur in different parts of the country. *Capensis* occurs to the south of the country in the Cape, with *scutellata* in the rest of the country countries to the north. Where they hybridise, *capensis* acts as a parasite, producing females from unfertilized eggs, effectively causing the demise of *scutellata* workers(South African National Biodiversity Institute 2015). There is now the possible threat that, as crop distributions move in response to climate change, that hives will be moved into different areas, which could worsen the problem of hybridisation between the two species. It might be that movements of other managed pollinators could also cause hybridisation problems in other parts of the globe. | South African National Biodiversity Institute. 2015. *Towards a national strategy for sustaining managed pollintion in South Africa*. Pretoria. | |
| 26 | Socioeconomic drivers of change in flowering crops: unpredictable outcomes | Agriculture dominates land use (Tilman et al. 2001) and, therefore, agricultural practices are thought to be important in dictating pollinator populations (e.g. Vanbergen et al. 2013). Flowering crops can provide rich resources in terms of pollen and nectar, giving incentives to beekeepers to maintain honey bees (*Apis mellifera*) for honey production and promoting populations of some wild bees (Herrmann et al. 2007; but see Westphal et al. 2009). Though the pollination benefits of flowering crops – through promoting pollinators – in enhancing pollination of other crops and wild plants is equivocal (via competition for pollinators: Holzschuh et al. 2011; via promotion of pollinator numbers and pollination: Holzschuh et al. 2013; Riedinger et al. 2014; Rundlöf et al. 2014), changes in socioeconomics may impact agricultural practices in two important ways. First, changes in political support for and economic demand for biofuels could markedly increase or decrease those mass flowering crops that yield biofuels. Second, increasing wealth of developing national could lead to demand for a meat-rich diet, promoting farming for livestock at the expense of flowering crops and pollination. | Herrmann F, Westphal C, Moritz RFA, Steffan-Dewenter I (2007) Genetic diversity and mass resources promote colony size and forager densities of a social bee (*Bombus pascuorum*) in an agricultural landscape. *Molecular Ecology* 16, 1167-1178.  Holzschuh A, Dormann CF, Tscharntke T, Steffan-Dewenter I (2011) Expansion of mass-flowering crops leads to transient pollinator dilution and reduced wild plant pollination. *Proceedings of the Royal Society B: Biological Sciences* 278, 3444-3451.  Holzschuh A, Dormann C, Tscharntke T, Steffan-Dewenter I (2013) Mass-flowering crops enhance wild bee abundance. *Oecologia* 172, 477-484.  Riedinger V, Renner M, Rundlöf M, Steffan-Dewenter I, Holzschuh A (2014) Early mass-flowering crops mitigate pollinator dilution in late-flowering crops. *Landscape Ecology* 29, 425-435.  Rundlöf M, Persson AS, Smith HG, Bommarco R (2014) Late-season mass-flowering red clover increases bumble bee queen and male densities. *Biological Conservation* 172, 138-145.  Tilman D, et al. (2001) Forecasting agriculturally driven global environmental change. *Science* 292, 281-284.  Vanbergen AJ, Initiative atIP (2013) Threats to an ecosystem service: pressures on pollinators. *Frontiers in Ecology and the Environment* 11, 251-259.  Westphal C, Steffan-Dewenter I, Tscharntke T (2009) Mass flowering oilseed rape improves early colony growth but not sexual reproduction of bumblebees. *Journal of Applied Ecology* 46, 187-193 | |
| 27 | Benefits to pollinators from water quality protection | The European Water Framework Directive in Europe and similar regulation in other parts of the world has greatly increased the use of buffer strips along water courses. These buffer strips can provide really important resources for pollinators if managed correctly (Cole *et al* 2015), so there is a strong synergy between water and pollinator policy that has not been fully exploited. Increasing evidence of this is expected over the coming years, and could lead to a substantial enhancement of floral resources in agricultural landscapes. | Cole, L. J., Brocklehurst, S., Robertson, D., Harrison, W., & McCracken, D. I. (2015). Riparian buffer strips: Their role in the conservation of insect pollinators in intensive grassland systems. *Agriculture, Ecosystems & Environment*, 211, 207-220. doi:http://dx.doi.org/10.1016/j.agee.2015.06.012 | |
| 28 | Treatments for managed honeybee bacterial diseases using phage therapy | American Foul Brood, caused by Paenibacillus larvae is the most serious bacterial disease affecting honey bee brood. American foulbrood is particularly problematic, as its spores can withstand desiccation and remain viable for decades. Bacteriophages are viruses that infect and replicate within bacteria. Bacteriophages are common and diverse and are usually found wherever there are bacterial hosts. Bacteriophages have an advantage over antibiotics because they are very specific to their hosts, targeting only one of a few bacterial strains, whereas antibiotics are far less specific and often kill beneficial bacteria in addition to the target bacteria. As bacteria evolve resistance to the phage, the phage evolves in response (<http://www.phagetherapycenter.com/pii/PatientServlet?command=static_phagetherapy&language=0>), so use of bacteriophages should be less susceptible to evolution of resistance in bacteria. Furthermore, antibiotics are metabolized and eliminated from the body, whereas bacteriophages replicate at the site of the infection, along with the bacteria, so the phage does not have to be administered as often as antibiotics might. Most work on bacteriophages is carried out in research on human health, but recent work in the laboratory suggests phage therapy might work for American Foulbrood disease, too (Ghorbani-Nezami et al. 2015), although this now needs to be tested in the field. Risks posed by bacteriophage therapy would also require investigation. | Ghorbani-Nezami, S., LeBlanc, L., Yost, D.G., & Amy, P.S. 2015. Phage Therapy is Effective in Protecting Honeybee Larvae from American Foulbrood Disease. *Journal of Insect Science* 15: 84 | |
| 29 | Novel pathogens: a threat to many bee species and pollination | Emerging pathogens are, by definition, a major health issue for humans and domestic plants and animals as well as being a threat to wildlife (Daszak et al. 2000; Woolhouse et al. 2005). RNA-viruses in particular are prominent among emerging pathogens (Cleaveland et al. 2001) because of their genome variability. Honey bees (*Apis mellifera*) suffer from a number of pathogenic RNA-viruses, especially deformed wing virus (DWV; Martin et al. 2012) that are probably causal in elevated rates of colony mortality (McMahon et al. in review). Viral recombination has been suggested as one mechanism generating genome variability and a more virulent form of DWV (Moore et al. 2011; Ryabov et al. 2014). This and other viruses of the honey bee are shared with wild bees, with the weight of evidence suggesting transmission is from honey bees to wild bees (Fürst et al. 2014; McMahon et al. 2015). Novel pathogens, particularly novel RNA viruses and novel variants of known RNA viruses, represent a future threat to honey bees. Through their emergence in this commonly managed pollinator, they may readily spread to other pollinator species and lead to future catastrophic declines in pollinators and pollination. | Cleaveland S, Laurenson MK, Taylor LH (2001) Diseases of humans and their domestic mammals: pathogen characteristics, host range and the risk of emergence. *Philosophical Transactions of the Royal Society of London B: Biological Sciences* 356, 991-999.  Daszak P, Cunningham AA, Hyatt AD (2000) Emerging infectious diseases of wildlife - threats to biodiversity and human health. *Science* 287, 443-449.  Fürst MA, McMahon DP, Osborne JL, Paxton RJ, Brown MJF (2014) Disease associations between honeybees and bumblebees as a threat to wild pollinators. *Nature* 506, 364-366.  Martin SJ, Highfield AC, Brettell L*, et al.* (2012) Global honey bee viral landscape altered by a parasitic mite. *Science* 336, 1304-1306.  McMahon DP, Fürst MA, Caspar J*, et al.* (2015) A sting in the spit: widespread cross-infection of multiple RNA viruses across wild and managed bees. *Journal of Animal Ecology* 84, 615-624.  McMahon DP, Natsopoulou M, Doublet V*, et al.* (in revision) More than a common cold: an emerging virus genotype drives elevated honeybee loss. *Nature Communications*.  Moore J, Jironkin A, Chandler D*, et al.* (2011) Recombinants between Deformed wing virus and Varroa destructor virus-1 may prevail in *Varroa destructor*-infested honeybee colonies. *Journal of General Virology* 92, 156-161.  Ryabov EV, Wood GR, Fannon JM*, et al.* (2014) A virulent strain of Deformed Wing Virus (DWV) of honeybees (*Apis mellifera*) prevails after *Varroa destructor*-mediated, or *in vitro*, transmission. *PLOS Pathogens* 10, e1004230.  Woolhouse MEJ, Haydon DT, Anita R (2005) Emerging pathogens: the epidemiology and evolution of species jumps. *Trends in Ecology and Evolution* 20, 238-244 | |
| 30 | Pollinators as Pathways for Pathogens | While pollinators are required for the production of many crops, these pollinators can also transmit diseases in the process. Managed pollinators can pass disease agents to more susceptible wild pollinators, affecting the wider pollination network. Pollinators can also play a role in the transmission of plant pathogens, including bacteria and viruses (McArt et al 2014; Pattemore et al 2014; Fürst et al 2014). Crop industries concerned about pollinator spread of disease could insist on restrictions on movements of pollinators, such as the case in New Zealand when honeybee hive movements were restricted to minimise the risk of spreading the bacterial pathogen *Pseudomona syringae* pv. *Actinidae* (Kiwifruit Vine Health 2014). This may provide an opportunity to promote and prioritise the use of local wild pollinators rather than managed pollinator species. | McArt S.H., Koch H., Irwin R.E., Adler L.S. (2014) Arranging the bouquet of disease: floral traits and the transmission of plant and animal pathogens. *Ecology Letters* 17: 624–636  Pattemore D.E., Goodwin R.M., McBrydie H.M., Hoyte S.M., Vanneste J.L. (2014) Evidence of the role of honey bees *(Apis melifera)* as vectors of the bacterial plant pathogen *Pseudomonas syringae. Australasian Plant Pathology* 43: 571-575.  Fürst M.A., McMahon D.P., Osborne J.L., Paxton R.J., Brown M.J.F. (2014) Disease associations between honeybees and bumblebees as a threat to wild pollinators. *Nature* 506:364-366.  Kiwifruit Vine Health (2014) KVH Protocol: Pollination with Bees, 19 August 2014, http://www.kvh.org.nz/vdb/document/94627 | |
| 31 | Reductions in pollinator species richness may drive epidemics | Species rich host communities support lower parasite abundance than species poor communities (Civitello et al. 2015). Species richness is believed to inhibit parasites through the dilution effect, including mechanisms such as population regulation of susceptible hosts, or interfering with transmission (Ostfeld and Keesing 2012). Pollinator declines are characterized by reductions in species richness, with a small number of apparently robust species surviving in anthropogenically impacted habitats (Kleijn et al. 2015). Given that emerging parasites already threaten wild pollinator assemblages (Fürst et al. 2014) and that populations of once abundant and widespread species can rapidly collapse across their range (Cameron et al. 2011), this suggests that reductions in species richness may lead to parasite epidemics in wild pollinator assemblages that could drive further rapid declines. | Cameron SA (2011) Patterns of widespread decline in North American bumble bees. *PNAS* 108:662-667  Civitello DJ, et al. (2015) Biodiversity inhibits parasites: Broad evidence for the dilution effect. *PNAS* 112:8667-8671  Fürst MA, et al. (2014) Disease associations between honeybees and bumblebees as a threat to wild pollinators. *Nature* 506:364-366  Kleijn D, et al. (2015) Delivery of crop pollination services is an insufficient argument for wild pollinator conservation. *Nature Comm* 6:7414  Ostfeld RS, Keesing F (2012) Effects of host diversity on infectious disease. *Ann Rev Ecol Evol Syst* 43:157-182 | |
| 32 | Honeybee viruses | The most frequently reported honeybee viruses are the Deformed Wing Virus (DWV), the Sacbrood Virus (SBV), the Chronic Bee Paralysis Virus (CBPV), the Black Queen Cell Virus (BQCV), and the viruses belonging to the Acute-Kashmir-Israeli complex. Although the use of RNAi has been proposed as treatment means (Chen et al., 2014; Maori et al., 2009), it must be recognized that to date only health management practices (isolation or destruction of the infected colony, frame changes, and / or renewal of the queen) can slow the spread of highly contagious viral diseases. Viral diseases do not appear to cause significant *acute* colony losses. Although the number of lost individuals can be very important in some cases (Ribière et al., 2009), the bee colonies usually recover to a normal activity over time. However, recent studies shed new light on the links between viruses with other stress, particularly vectors (Varroa mites) and pesticides. Many studies have shown most important health effects on honeybees associating virus with other stresses: *N. apis* and CBPV (Toplak et al., 2015), *V. destructor* and DWV (Mondet et al., 2014), and neonicotinoids and DWV (Di Prisco and al., 2013).  The challenges of the coming years will be to improve the diagnosis of viral diseases including measuring the virus replication rate in honeybees as the number of viruses per bee is an indicator of honeybee health disorder. | Chen YP, Pettis JS, Corona M, Chen WP, Li CJ, Spivak M, Visscher PK, DeGrandi-Hoffman G, Boncristiani H, Zhao Y, vanEngelsdorp D, Delaplane K, Solter L, Drummond F, Kramer M, Lipkin WI, Palacios G, Hamilton MC, Smith B, Huang SK, Zheng HQ, Li JL, Zhang X, Zhou AF, Wu LY, Zhou JZ, Lee ML, Teixeira EW, Li ZG, Evans JD. 2014. Israeli acute paralysis virus: epidemiology, pathogenesis and implications for honey bee health. *PLoS Pathog*. 2014 Jul 31;10(7):e1004261.  Maori E, Paldi N, Shafir S, Kalev H, Tsur E, Glick E, Sela I. 2009. IAPV, a bee-affecting virus associated with Colony Collapse Disorder can be silenced by dsRNA ingestion. *Insect Mol Biol*. 2009 Feb;18(1):55-60.  Ribière M, Olivier V, Blanchard P. 2009. Chronic bee paralysis: a disease and a virus like no other? *J Invertebr Patho*l. 2010 Jan;103 Suppl 1:S120-31.  Toplak I, Jamnikar Ciglenečki U, Aronstein K, Gregorc A. 2013. Chronic bee paralysis virus and *Nosema ceranae* experimental co-infection of winter honey bee workers (*Apis mellifera* L.). *Viruses*. Sep 19;5(9):2282-97.  Di Prisco G, Cavaliere V, Annoscia D, Varricchio P, Caprio E, Nazzi F, Gargiulo G, Pennacchio F. 2013. Neonicotinoid clothianidin adversely affects insect immunity and promotes replication of a viral pathogen in honey bees. *Proc Natl Acad Sci U S A*. 2013 Nov 12;110(46):18466-71.  Mondet F, de Miranda JR, Kretzschmar A, Le Conte Y, Mercer AR. 2014. On the front line: quantitative virus dynamics in honeybee (*Apis mellifera* L.) colonies along a new expansion front of the parasite *Varroa destructor*. *PLoS Pathog*. 21;10(8):e1004323. | |
| 33 | Bacterial diseases  American foulbrood & European foulbrood | American foulbrood (AFB), a contagious disease caused by the spore-forming bacterium *Paenibacillus larvae*, is the most deleterious disease on honeybee brood. The bacterium is distributed worldwide (Matheson, 1993). The spores are resistant to antibiotics (Genersch and Otten, 2003), heat and drought and can remain infective for more than 35 years (Hasemann, 1961). Four genotypes of *P. larvae* have been identiﬁed (Genersch et al., 2006). The genotypes vary in phenotypical and virulence characteristics (Fünfhaus et al., 2013; Genersch et al., 2005; Genersch et al., 2006; Neuendorf et al., 2004; Poppinga et al., 2012; Rauch et al., 2009).  To identify virulence factors/mechanisms, to set up highly sensitive methods (Rivière et al., 2013) allowing the early detection of *P. larvae* and to study the variability and the biogeography of the bacterium (Morrissey et al., 2014) are as many objectives to reach in order to better control the disease.  European foulbrood (EFB), caused by the bacterium *Melissococcus plutonius*, is a serious disease of honey bee (*Apis mellifera*) brood. The bacterium is widely distributed and its infection can lead to the loss of the host colony (Forsgren et al., 2013). EFB is usually considered as a minor cause of concern compared to AFB. However, an increase of outbreaks has been recently reported in United Kingdom and Switzerland (Wilkins et al., 2007; Roetschi et al., 2008). It has also been demonstrated that various *M. plutonius* strains can show various virulence characteristics *in vitro* (Arai *et al*., 2012). During the past few years several studies have been conducted in order to highlight the epidemiological, physiological and virulence characteristics of the bacteria (Budge et al., 2010; Budge et al., 2014; Takamatsu et al., 2013; Takamatsu et al., 2014). | Fünfhaus A, Poppinga L, and Genersch E (2013) Identiﬁcation and characterization of two novel toxins expressed by the lethal honey bee pathogen *Paenibacillus larvae*, the causative agent of American foulbrood: two novel *Paenibacillus larvae* toxins. *Environ Microbiol* 15: 2951–2965  Genersch E, and Otten C (2003) The use of repetitive element PCR ﬁngerprinting (rep-PCR) for genetic subtyping of German ﬁeld isolates of *Paenibacillus larvae* subsp. *larvae*. *Apidologie* 34: 195–206  Genersch E, Ashiralieva A, and Fries I (2005) Strain- and genotype-speciﬁc differences in virulence of *Paenibacillus larvae* subsp. *larvae*, a bacterial pathogen causing American foulbrood disease in honeybees. *Appl Environ Microbiol* 71: 7551–7555  Genersch E, Forsgren E, Pentikäinen J, Ashiralieva A, Rauch S, Kilwinski J, and Fries I (2006) Reclassiﬁcation of *Paenibacillus larvae* subsp. *pulvifaciens* and *Paenibacillus larvae* subsp. *larvae* as *Paenibacillus larvae* without subspecies differentiation. *Int J Syst Evol Microbiol* 56: 501–511  Hasemann L (1961) How long can spores of American foulbrood live? *Am Bee J* 101: 298–299  Matheson A (1993) World bee health report. *Bee World* 74:176–212  Morrissey BJ, Helgason T, Poppinga L, Fünfhaus A, Genersch E, Budge G (2014) Biogeography of *Paenibacillus larvae*, the causative agent of American foulbrood, using a new multilocus sequence typing scheme *Environmental Microbiology* (2015) 17(4), 1414–1424  Neuendorf S, Hedtke K, Tangen G, and Genersch E (2004) Biochemical characterization of different genotypes of *Paenibacillus larvae* subsp. *larvae*, a honey bee bacterial pathogen. *Microbiology* 150: 2381–2390  Poppinga L, Janesch B, Fünfhaus A, Sekot G, Garcia-Gonzalez E, Hertlein G, Hedtke K, Schaffer C, Genersch E (2012) Identiﬁcation and functional analysis of the S-layer protein SplA of *Paenibacillus larvae*, the causative agent of American foulbrood of honey bees. *PLoS Pathog* 8: e1002716  Rauch S, Ashiralieva A, Hedtke K, and Genersch E (2009) Negative correlation between individual-insect-level virulence and colony-level virulence of *Paenibacillus larvae*, the etiological agent of American foulbrood of honeybees. *Appl Environ Microbiol* 75: 3344–3347  Rivière MP, Ribière M, Chauzat MP (2013) Recent molecular biology methods for foulbrood and nosemosis diagnosis. *Rev. sci. tech. Off. int. Epiz.*, 2013, 32 (3), 885-892  Arai R, Tominaga K, Wu M, Okura M, Ito K, Onishi H, Osaki M, Sugimura Y, Yoshiyama M, Okamura N, Takamatsu D (2012) Diversity of *Melissococcus plutonius* from Honeybee Larvae in Japan and Experimental Reproduction of European Foulbrood with Cultured Atypical Isolates. *PLoS ONE* 7(3): e33708. doi:10.1371/journal.pone.0033708  Budge GE, Barrett B, Jones B, Pietravalle S, Marris G, Chantawannakul P, Thwaites R, Hall J, Cuthbertson AGS, Brown MA (2010) The occurrence of *Melissococcus plutonius* in healthy colonies of *Apis mellifera* and the efficacy of European foulbrood control measures. *J Invertebr Pathol* 105:164–170  Forsgren E, Budge GE, Charrière J-D, Hornitzky MAZ (2013) Standard methods for European foulbrood research. *J Apic Res* 52: 1–14  Roetschi A, Berthoud,RolfKuhn H, Imdorf A (2008) Infection rate based on quantitative real-time PCR of Melissococcus plutonius, the causal agent of European oulbrood, in honeybee colonies before and after apiary sanitation. *Apidologie* 39 (2008) 362–371  Takamatsu D, Arai R, Miyoshi-Akiyama T, Okumura K, Okura M, Kirikae T et al. (2013). Identification of mutations involved in the requirement of potassium for growth of typical *Melissococcus plutonius* strains. *Appl Environ Microbiol* 79: 3882–3886  Takamatsu D, Morinishi K, Arai R, Sakamoto A, Okura M, Osaki M (2014) Typing of *Melissococcus plutonius* isolated from European and Japanese honeybees suggests spread of sequence types across borders and between different *Apis* species. *Veterinary Microbiology* 171, 221–226  Wilkins S, Brown MA and Cuthbertson, AGS (2007) Perspective: the incidence of honey bee pests and diseases in England and Wales. *Pest Manage. Sci*. 63, 1062-1068 | |
| 34 | New emerging diseases: Small hive beetle *Aethina tumida* | Small hive beetles (SHB) are generalists native to sub-Saharan Africa and reproduce in association with honeybees, bumblebees, stingless bees, fruits and meat. SHB has recently become an invasive species and introductions have been recorded from America, Australia, Europe and Asia since 1996. While SHB are usually considered a minor pest in Africa, they can cause significant damage to social bee colonies in their new ranges. Potential reasons for differential impact include differences in bee behavior, climate and release from natural enemies. However, data for hosts other than honey bees are largely lacking, thereby creating an urgent demand to evaluate their impact after pathogen shifts. The potential for an invasion meltdown scenario is considerably high, especially in tropical areas fostering SHB reproduction (Central and South America, South East Asia, Australasian region). | Neumann et al. (2016) Quo vadis *Aethina tumida*? Biology and control of small hive beetles. *Apidologie*, in press. | |
| 35 | New emerging diseases: *Tropilaelaps* spp. | Historically ectoparasites of the native Giant honey bee, *Apis dorsata*, the mites *Tropilaelap*s spp. have switched hosts to the introduced western honey bee *Apis mellifera* throughout much of Asia. Few data regarding lethal and sub-lethal effects of *T. mercedesae* on *A. mellifera* exist, despite its similarity to the devastating mite *Varroa destructor*. Recent reports from Asian beekeepers and some data suggest that at least *T. mercedesae* is a devastating, yet completely understudied, parasite to the economically important *A. mellifera* honey bee. Since mites of the genus *Tropilaelap*s spp. replicate faster than *V. destrcutor*, but are highly dependent upon brood, climate change with reduced brood-free periods may foster the impact of this mite genius, thereby creating even more demand to study both its biology and control. | Khongphinitbunjong et al (2015) The ectoparasitic mite *Tropilaelaps mercedesae* reduces western honey bee, *Apis mellifera*, longevity and induces clinical symptoms. J Inv Pathol, under consideration. | |
| 36 | Varroa 2.0 | *Varroa destructor* is the worst emergent disease to have impacted *A. mellifera* in last 100 years. The mite ‘jumped species’ from *A. cerana* to *A. mellifera* to become *Varroa destructor* most likely in the 1960s in the Philippines. Now a new host shift seems to be occurring. In the highlands of Papua New Guinea *V. jacobsoni* has been found reproducing on both *A. mellifera* and *A. cerana*. Previously, *V. jacobsoni* was thought to be restricted to *A. cerana* and not a threat to *A. mellifera*, but since the two *Apis* species have been sympatric in Papua New Guinea since 1987 a new and completely independent shift of *Varroa* from *A. cerana* onto *A. mellifera* seems to be occurring. We do not know to what degree this will complicate battling *Varroa* infections in *A. mellifera*, but it is unlikely to help. | Roberts J.M.K., Anderson D.L. & Tay W.T. 2015 Multiple host shifts by the emerging honeybee parasite *Varroa jacobsoni*. *Molecular Ecology* 23 2379-2391 | |
| 37 | Infection with *Nosema* spp. | *Nosema ceranae* is a microsporidian parasite described from the Asian honey bee, *Apis cerana* (Fries et al. 1988). The parasite is cross-infective with the European honey bee, *Apis mellifera*. It is not known when or where *N. ceranae* first infected European bees, but *N. ceranae* has probably been infecting European bees for at least two decades (Klee et al. 2007; Paxton et al. 2007). N. ceranae appears to be replacing Nosema apis, at least in some populations of European honey bees (Fries, 2010). Cross-infection experiments using both N. ceranae and N. apis in both A. cerana and A. mellifera demonstrated that both parasites were cross-infective across hosts, but that N. ceranae developed better in A. mellifera compared to N. apis in A. cerana (Fries and Feng, 1995; Fries, 1997). The infectivity of N. ceranae to A. mellifera is not surprising since many microsporidia exploit multiple hosts. Among the factors influencing the infectiousness of *Nosema spp.* in honeybees, diets and temperature changes have been specifically studied in recent years. To overcome the lack of food due to various reasons (development of monocultures, climate disorder), beekeepers use pollen substitute diets. Some studies clearly demonstrated that some supplemented pollens enhanced the infectivity of microsporidian parasites and thereby increased the weakness of the colonies (Porrini et al. 2011). The physiological characteristic of the midgut and the physical characteristic of the artificial products were directly implicated in the Nosema spp. levels. The question of the difference in virulence between the two species of Nosema remains open. For N. ceranae no specific colony level symptoms of infection have been described. In Spain, infected colonies have been associated with gradual depopulation, higher autumn and winter colony death and a decrease in honey production (Higes et al., 2008). These results were the subject of controversial discussions and experiments (Higes et al. 2013). Others studies are currently conducted in order to explore the change in virulence observed in different *N. ceranae* populations (Dussaubat et al. 2013, Van der Zee 2014). | Dussaubat, C., Sagastume, S., Gómez-Moracho, T., Botías, C., García-Palencia, P., Martín-Hernández, R., et al., 2013. Comparative study of *Nosema ceranae* (Microsporidia) isolates from two different geographic origins. *Vet Microbiol* 162: 670–678.  Fries, I., 1988. Infectivity and multiplication of *Nosema apis* Z. in the ventriculus of the honey bee. *Apidologie* 19, 319–328.  Fries, I., Feng, F., 1995. Crossinfectivity of *Nosema apis* in *Apis mellifera* and *Apis cerana*. In: Proceedings of the Apimondia 34th International Apicultural Congress. Bucharest, Romania, pp. 151–155.  Fries, I., 1997. Protozoa. In: Morse, R.A., Flottum, K. (Eds.), Honey Bee Pests, Predators and Diseases, third ed. A.I. Root Company, Medina, Ohio, USA, pp. 59–76.  Fries, I., 2010. *Nosema ceranae* in European honey bees (*Apis mellifera*). *J Invertebr Pathol* 103 (Suppl.): S73–S79.  Higes, M., Martín-Hernandez, R., Botias, C., Bailon, E.G., Gonzales-Porto, A., Barrios, L., del Nozal, M.J., Palencia, P.G., Meana, A., 2008. How natural infection by *Nosema ceranae* causes honeybee colony collapse. *Environ. Microbiol*. 10, 2659–2669.  Higes, M., Meana, A., Bartolomé, C., Botías, C.,and Martín-Hernández, R. , 2013. *Nosema ceranae* (Microsporidia), a controversial 21st century honey bee pathogen. *Environ Microbiol Rep* 5: 17–29.  Klee, J., Besana, A., Genersch, E., Gisder, S., Nanetti, A., Tam, D.Q., Chinh, T.X., Puerta, F., Kryger, P., Message, D., Hatjina, F., Korpela, S., Fries, I., Paxton, R., 2007. Widespread dispersal of the microsporidium *Nosema ceranae*, an emergent pathogen of the western honey bee, *Apis mellifera*. *J. Invertebr. Pathol*. 96, 1–10.  Paxton, R.J., Klee, J., Korpela, S., Fries, I., 2007. *Nosema ceranae* has infected *Apis mellifera* in Europe since at least 1998 and may be more virulent than *Nosema apis*. *Apidologie* 38, 558–565.  Porrini, M.P., Sarlo E.G., Medici S.K., Garrido P.M., Porrini D.P., Damiani N., Eguaras M.J., 2011. *Nosema ceranae* development in *Apis mellifera*: influence of diet and infective inoculum. *Journal of Apicultural Research* 50(1): 35-41.  Van der Zee R., Pisa L.,Gómez-Moracho T., Sagastume S., García-Palencia P., Maside X., Bartolomé C., Martín-Hernández R. and Higes M., 2014. Virulence and polar tube protein genetic diversity of *Nosema ceranae* (Microsporidia) ﬁeld isolates from Northern and Southern Europe in honeybees (*Apis mellifera iberiensis*). *Environ Microbiol Rep* 4: 401-413 | |
| 38 | Co-exposure between pesticides and pathogens | Honeybees can host many pathogens (bacteria, virus, fungi, mites) and be simultaneously exposed to various sources of pesticides. Pesticides can be carried back to the hive by the bees, and be found as residues in many hive matrixes, such as pollen, nectar, bee bread, honey or beeswax (Chauzat et al., 2011, Mullin et al. 2010).  The co-exposures lead to interaction between pathogens, between pesticides and between pathogens and pesticides. Pesticides interact amongst each other in the honeybee. The acaricide tau-fluvalinate, used to control *V. destructor*, has been found to decrease the topical LD_50_ of prochloraz (a fungicide) of a 100-fold (Johnson et al. 2013). Pathogens also interact with each other. A synergistic effect has been demonstrated between *Nosema ceranae* and the BQCV virus on honeybee mortality (Doublet et al. 2014). Co-exposures between pesticides and pathogens have also been studied, especially between *N. ceranae* and neonicotinoids. Synergistic effects between *N. ceranae* and imidacloprid (Alaux et al. 2010), and with fipronil and thiacloprid (Vidau et al. 2011) have been observed on honeybee mortality. Combined effects of pesticides and honeybee viruses also demonstrated synergistic effect on honeybee mortality and on the inhibition of the immune response when honeybees were exposed to clothianidin or imidacloprid and DWV at various doses (Di Prisco et al. 2013). The replication of the BQCV virus was enhanced when honeybee were previously exposed to sublethal doses of thiacloprid (Doublet et al. 2014). In conclusion, co-expositions to both stress factors should be taken as a rule rather than an exception in honeybee research. | Alaux, C. et al., 2010. Interactions between *Nosema* microspores and a neonicotinoid weaken honeybees (*Apis mellifera*). *Environmental Microbiology*, 12(3), pp.774–782.  Chauzat, M.-P. et al., 2011. An assessment of honeybee colony matrices, *Apis mellifera* (Hymenoptera Apidae) to monitor pesticide presence in continental France. *Environmental Toxicology and Chemistry*, 30(1), pp.103–111.  Doublet, V. et al., 2014. Bees under stress: sublethal doses of a neonicotinoid pesticide and pathogens interact to elevate honey bee mortality across the life cycle. *Environmental Microbiology*  Johnson, R.M. et al., 2013. Acaricide, Fungicide and Drug Interactions in Honey Bees (*Apis mellifera*). Plos One, 8(1)  Mullin, C.A. et al., 2010. High levels of miticides and systemic agrochemicals in North American beehives: Implications for honey bee health. Abstracts of Papers of the American Chemical Society, 239.  Di Prisco, G. et al., 2013. Neonicotinoid clothianidin adversely affects insect immunity and promotes replication of a viral pathogen in honey bees. *Proceedings of the National Academy of Sciences of the United States of America*, 110(46), pp.18466–18471.  Vidau, C. et al., 2011. Exposure to Sublethal Doses of Fipronil and Thiacloprid Highly Increases Mortality of Honeybees Previously Infected by *Nosema ceranae*. *Plos One*, 6(6). | |
| 39 | Sanitary and genetic issues raised by international trade and globalization | Honeybee colony losses and the need of pollination have lead to the intensification of commercial exchanges of kept pollinators (including bumble bees) worldwide. These movements bring some sanitary issues related to the introduction of new pathogenic agents or new strains to which local pollinators may not be adapted. The yellow hornet, *Vespa velutina*, a predator native from Asia, was discovered in France in 2004 and is now spreading across Europe (Rome et al. 2015). The presumed origin of this introduction is the importation of pottery from China. The small hive beetle (Ae*thina tumida*), a pest of the African honeybees, has been introduced in several countries (and recently Italy) causing damages to beekeeping (Neumann and Elzen, 2004; OIE, 2013). *Varroa destructor*, a parasitic mite originally endemic to Asia, is now present all over the world, inducing colony weakness and losses. The virulence of the different pathogen strains circulating around the world need to be better understood to evaluate their impact on honeybee health.  The question of genetic is also raised by the introduction of non-endemic species, sub-species, breed or strains of pollinators. Introduced pollinators can compete with local species and/or hybridize with them. Swarms or feral colonies are not always detected and destroyed in containers and can reach new area by this mean (see example of A. cerana in Australia). | Neumann, P., Elzen, P.J., 2004. The biology of the small hive beetle (*Aethina tumida*, Coleoptera: Nitidulidae): Gaps in our knowledge of an invasive species. *Apidologie* 35, 229-247.  Rome, Q., Villemant, C. Le Frelon asiatique *Vespa velutina* - Inventaire national du Patrimoine naturel. In: Muséum national d’Histoire naturelle [Ed]. Internet website: <http://frelonasiatique.mnhn.fr>; Webpage, assessed on 13 august 2015 | |
| 40 | Climate change: altering pathogen epidemiology to the detriment of pollinators | For insects, climate change is predicted to have greatest negative impact in the tropics, where many insect species are near their upper thermal tolerance, but to have little negative or even a positive impact in temperate regions of the world (Deutsch et al. 2008). Though climate change might seem to spell well for EU insect pollinators, their parasites (pests and pathogens) may benefit to a greater extent. The exotic and emergent gut parasite *Nosema ceranae*, common in honey bees (*Apis mellifera*) and infective in *Bombus* spp. (Fürst et al. 2014), predominates in hot climates and theoretical modelling suggests that its competitive advantage is enhanced under climate warming (Natsopoulou et al. 2015). Climate warming may also increase the duration that brood is present in honey bee colonies, increasing the build-up of *Varroa destructor* mites that are major transmitters of pathogenic viruses between honey bees (Rosenkranz et al. 2010). Increasing pathogen loads of honey bees will lead to a greater force of infection of – pathogen spill-over to – wild bee species (e.g. Fürst et al. 2014; McMahon et al. 2015). | Deutsch CA, Tewksbury JJ, Huey RB*, et al.* (2008) Impacts of climate warming on terrestrial ectotherms across latitude. *Proceedings of the National Academy of Sciences* 105, 6668-6672.  Fürst MA, McMahon DP, Osborne JL, Paxton RJ, Brown MJF (2014) Disease associations between honeybees and bumblebees as a threat to wild pollinators. *Nature* 506, 364-366.  McMahon DP, Fürst MA, Caspar J*, et al.* (2015) A sting in the spit: widespread cross-infection of multiple RNA viruses across wild and managed bees. *Journal of Animal Ecology* 84, 615-624.  Natsopoulou ME, McMahon DP, Doublet V, Bryden J, Paxton RJ (2015) Interspecific competition in honeybee intracellular gut parasites is asymmetric and favours the spread of an emerging infectious disease. *Proceedings of the Royal Society of London B: Biological Sciences* 282, 20141896.  Rosenkranz P, Aumeier P, Ziegelmann B (2010) Biology and control of *Varroa destructor*. *Journal of Invertebrate Pathology* 103, S96-S119. | |
| 41 | Changes in nutritional value of plants as a consequence of elevated atmospheric CO2 and pollution associated with human activities | Increased concentrations of CO2 in the atmosphere translates into faster plant growth, but this is usually accompanied by higher C:N ratios in plant tissue, depending on soil nitrogen (Griffin et al. 2009). Nectar quantities may increase because C rich compounds will be easier to produce, but they may have lower amino acid concentrations. Pollen, too may have lower protein content. Pollen is the main protein source for many pollinators, particularly bees (Gilbert 1972 in Yeamans et al 2014). Butterfly fed nectar with higher amino acid concentrations laid more eggs than those that had not been fed nectar containing amino acids, although this effect was not shown for butterflies which had been raised on nitrogen-rich food (Mevi-Schutz & Erhardt 2005). Plant material containing higher C:N ratios had more damage from butterfly larvae (Lincoln et al. 1986), presumably to meet their protein requirements. Changes in temperature and carbon dioxide also affect plant signalling and pollen value (Parachnowitsch & Manson 2015), which should in turn affect pollination. A recent study assessing the effects of nitrogen deposition, increased CO_2_ concentrations and increased temperature found that these influences affected flower morphology and phenology, sex ratios and nectar chemistry, changing the attractiveness of nectar, with bumblebees being more attracted to nectar that was detrimental to their health (Hoover et al. 2012). There seems large scope for research into the combined effects of these drivers associated with climate change. | Griffin, J.N., Méndez, V., Johnson, A.F., Jenkins, S.R., & Foggo, A. 2009. Functional diversity predicts overyielding effect of species combination on primary productivity. *Oikos* 118: 37–44.  Hoover, S.E.R., Ladley, J.J., Shchepetkina, A.A., Tisch, M., Gieseg, S.P., & Tylianakis, J.M. 2012. Warming, CO2, and nitrogen deposition interactively affect a plant-pollinator mutualism. *Ecology Letters* 15: 227–234.  Lincoln, D., Couvet, D., & Sionit, N. 1986. Response of an insect herbivore to host plants grown in carbon dioxide enriched atmospheres. *Oecologia* 69: 556–560Mevi-Schutz, J., & Erhardt, A. 2005. Amino acids in nectar enhance butterfly fecundity: a long-awaited link. *The American naturalist* 165: 411–419.  Parachnowitsch, A.L., & Manson, J.S. 2015. The chemical ecology of plant-pollinator interactions: recent advances and future directions. *Current Opinion in Insect Science* 8: 4–10  Yeamans, R.L., Roulston, T.H., & Carr, D.E. 2014. Pollen quality for pollinators tracks pollen quality for plants in *Mimulus guttatus*. *Ecosphere* 5: article 91 | |
| 42 | Increasing frequency of heatwaves and droughts may drive pollinator declines | The most recent IPCC report on climate change states that an increase in the frequency and duration of heatwaves is very likely, and that mean precipitation is likely to decrease in mid-latitude and sub-tropical dry regions (IPCC 2013). Such changes in climate will reduce food availability to pollinators, as drought stress reduces nectar production (Carroll et al. 2001). Ad hoc field observations suggest that such weather patterns may impact bumble bee populations (Rasmont and Iserbyt 2012), and they are likely to have broader consequences for pollinators more generally, as nectar and the sugar it contains is the main energy source for pollinators. These predictions provide the opportunity to consider how to future-proof pollinator planting schemes, focusing on planting that contains drought-resistant species to provide nectar throughout extreme weather events. | Carroll AB, et al. (2001) Drought stress, plant water status, and floral trait expression in fireweed, *Epilobium angustifolium* (Onagraceae). *Am J Bot* 88:438-446  IPCC (2013) Summary for Policymakers. In: Climate Change 2013: The Physical Science Basis. Contribution of Working Group 1 to the Fifth Assessment Report of the Intergovernmental Panel on Climate Change. Stocher TF, et al. [eds]. Cambridge University Press, Cambridge, UK Rasmont P, Iserbyt S (2012) The bumblebees scarcity syndrome: are heat waves leading to local extinctions of bumblebees (Hymenoptera: Apidae: *Bombus*)? *Ann Soc Entomol Fr* 48:275-280 | |
| 42 | Extreme weather events vs. gradual climate change | Effects of gradual changes in recent or projected future climate on pollinators are increasingly well analysed (Kerr *et al.*, 2015, Rasmont *et al.*, 2015) but research on the impacts of extreme events such as heat waves and corresponding droughts is still in its infancy. Such climatic extremes can be expected to increase in frequency and intensity (Seneviratne *et al.*, 2014) and there is evidence that such extremes can lead to local extinctions of pollinators such as butterflies or bumblebees (Oliver *et al.*, 2015, Rasmont & Iserbyt, 2012). The impact of extreme events can be either direct by acting on the species’ physiology or indirect by generating resource bottlenecks (Maron *et al.*, 2015) and can even lead to the breakdown of plant-pollinator relationships (Harrison, 2000). The ability of population recovery and thus the consequences for ecosystem resilience are likely to depend on the frequency of the extreme events, the sensitivity of the species to the relevant environmental factor, species-specific life history traits (Ma *et al.*, 2015) and interacting landscape properties (Oliver *et al.*, 2015). Knowledge on the relative importance of extreme events in addition to the impacts of gradually changing conditions is of utmost importance to assess the consequences of climate change for pollinators and animal-pollinated plants. | Harrison RD (2000). Repercussions of El Nino: drought causes extinction and the breakdown of mutualism in Borneo. *Proceedings of the Royal Society B-Biological Sciences* 267: 911-915.  Kerr JT, Pindar A, Galpern P, Packer L, Potts SG, Roberts SM, Rasmont P, Schweiger O, Colla SR, Richardson LL, Wagner DL, Gall LF, Sikes DS, Pantoja A (2015). Climate change impacts on bumblebees converge across continents. *Science* 349: 177-180.  Ma G, Rudolf VHW, Ma CS (2015). Extreme temperature events alter demographic rates, relative fitness, and community structure. *Global Change Biology* 21: 1794-1808.  Maron M, Mcalpine CA, Watson JEM, Maxwell S, Barnard P (2015). Climate-induced resource bottlenecks exacerbate species vulnerability: a review. *Diversity and Distributions* 21: 731-743.  Oliver TH, Marshall HH, Morecroft MD, Brereton T, Prudhomme C, Huntingford C (2015). Interacting effects of climate change and habitat fragmentation on drought-sensitive butterflies. *Nature Clim. Change* advance online publication.  Rasmont P, Franzén M, Lecocq T, Harpke A, Roberts S, Biesmeijer JC, Castro L, Cederberg B, Dvorak L, Fitzpatrick Ú, Gonseth Y, Haubruge E, Mahé G, Manino A, Michez D, Neumayer J, Ødegaard F, Paukkunen J, Pawlikowski T, Potts S, Reemer M, Settele J, Straka J, Schweiger O (2015). Climatic Risk and Distribution Atlas of European Bumblebees. *BioRisk* 10: 1-236.  Rasmont P, Iserbyt S (2012). The Bumblebees Scarcity Syndrome: Are heat waves leading to local extinctions of bumblebees (Hymenoptera: Apidae: *Bombus*)? *Annales de la Societe Entomologique de France* 48: 275-280.  Seneviratne SI, Donat MG, Mueller B, Alexander LV (2014). No pause in the increase of hot temperature extremes. *Nature Clim. Change* 4: 161-163 | |
| 43 | Impact of climate change on plant-pollinator interactions | Climate change will lead to the generation of novel communities and thus change the interactions within them. These changed interactions in type and strength concern plant-pollinator interactions by changing patterns of spatial and temporal co-occurrence (Schweiger *et al.*, 2010). Less well understood are impacts of changes in the functional structure of plant and pollinator communities. For instance, climate change is likely to lead to a decline in the average body size of local bee communities (Franzén *et al.*, in prep.). Since body size is related to many functional aspects, such as flight distance or foraging behaviour, the character of pollination services might change considerably. However, a detailed understanding of potential consequences of these changes is still lacking. | Franzén M, Biesmeijer JC, Heikkinen RK, Harpke A, Krauss J, Kuhlmann M, De Meulemeester T, Michez D, Öckinger E, Pauly A, Potts SG, Rasmont P, Roberts SPM, Settele J, Wiemers M, Schweiger O (in prep.). Size matters in a changing climate: differential responses of bees and butterflies.  Schweiger O, Biesmeijer JC, Bommarco R, Hickler T, Hulme PE, Klotz S, Kühn I, Moora M, Nielsen A, Ohlemuller R, Petanidou T, Potts SG, Pyšek P, Stout JC, Sykes MT, Tscheulin T, Vila M, Walther GR, Westphal C, Winter M, Zobel M, Settele J (2010). Multiple stressors on biotic interactions: how climate change and alien species interact to affect pollination. *Biological Reviews* 85: 777-795. | |
| 44 | Impact of climate change on pollinator-pollinator interactions | The stress gradient hypothesis predicts that abiotic conditions limit species distributions at the more extreme ends of the stress gradient (e.g. cold or dry conditions) while negative biotic interactions, such as competition, limit the range under more benign abiotic conditions (Bertness & Callaway, 1994). As a consequence, the amount and strength of competition is defined by the level of co-occurrence of potentially competing species. With climate warming, we expect pollinators to expand their ranges, e.g. by moving to higher altitudes (Kerr *et al.*, 2015) or latitudes (Pöyry *et al.*, 2009), and thus the area of co-occurrence can be increased which in turn increases the amount of competition among pollinators. Effects of increased competition, e.g. for floral or nesting resources, might be of most concern in areas where these resources are already highly limited and natural boundaries do not allow range adjustments of the less competitive species. However, another prediction of the stress gradient hypothesis is that along the stress gradient predominantly competitive interactions are gradually replaced by facilitative interactions (Bertness & Callaway, 1994). Indirect facilitation among pollinators can be suspected if the overall effects of pollinators increases plant population viability and abundance more than a single pollinator can do (Lever *et al.*, 2014). However, whether indirect facilitation outweighs that of direct competition especially under increased stressful conditions remains to be solved. One particular challenge to solve this issue would be to obtain community data at fine enough grains while having sufficient spatial extents to cover larger environmental gradients and to be able to disentangle otherwise highly collinear environmental factors. | Bertness MD, Callaway R (1994). Positive interactions in communities. *Trends in Ecology & Evolution* 9: 191-193.  Kerr JT, Pindar A, Galpern P, Packer L, Potts SG, Roberts SM, Rasmont P, Schweiger O, Colla SR, Richardson LL, Wagner DL, Gall LF, Sikes DS, Pantoja A (2015). Climate change impacts on bumblebees converge across continents. *Science* 349: 177-180.  Lever JJ, Van Nes EH, Scheffer M, Bascompte J (2014). The sudden collapse of pollinator communities. *Ecology Letters* 17: 350-359.  Pöyry J, Luoto M, Heikkinen RK, Kuussaari M, Saarinen K (2009). Species traits explain recent range shifts of Finnish butterflies. *Global Change Biology* 15: 732-743. | |
| 45 | Decline and eventual disappearance of bumblebees due to climate change | Recent analysis seems to show that bumblebee ranges are shrinking, rather than moving northwards due to climate change in the Northern hemisphere (Kerr *et al.* 2015). If this pattern continues over the long term, it could lead to extinction of the entire bumblebee clade. While a remote possibility, this has large consequences for pollination. Research is urgently needed to understand the mechanism behind the observed pattern and design appropriate mitigation mechanisms. | Kerr, J. T., Pindar, A., Galpern, P., Packer, L., Potts, S. G., Roberts, S. M., . . . Pantoja, A. (2015). Climate change impacts on bumblebees converge across continents. *Science*, 349(6244), 177-180. doi:10.1126/science.aaa7031 | |
| 46 | The impact of invasive alien commercial honeybees on native bees in Asia | Invasive alien pollinators rarely change overall pollinator abundance or diversity, but can have direct negative effects on particular native species. Some bee species introduced to provide apicultural or pollination services to agriculture can also disrupt native pollinator communities either by directly outcompeting indigenous insects for floral of nesting resources or by spreading pests and pathogens to which other pollinators are susceptible. *A. mellifera* has been introduced to various regions of Asia e.g. *A. mellifera* was introduced to Japan in 1877 (Arai 2012), China in 1893 (Yang, 2005) and Thailand in 1975 (Sanpa and Chantawannakul 2009). Their flexibility to adapt to different environments has made them popular in the beekeeping industry. During 1993-2013, Asia became the continent with the largest number of managed colonies in the world with 41% (35.7m colonies) followed by Europe (22%), Africa (21.7%), America (14.3%) and Oceania (1%), of a global population of 72.6 million colonies (FAO, 2014). But in recent years in China, the distribution area of Chinese *A. cerana* has reduced by over 75%, and population numbers reduced over 80%, possibly due to competition with *Apis mellifera*, accompanied by changes of plant biodiversity, loss of habitat, competition with non-native species (Yang, 2005; Ji et al., 2003). *Bombus terrestris* has been forbidden to be introduced into Japan, but it has just been introduced to China for pollination; it may reduce native *Bombus* species richness and abundance. China has the world’s highest bumblebee diversity, with about 125 of the 250 recognized species, which could could be at risk from imported *B. terrestris*（An, et al, 2014). | An, J., Huang, J., Shao, Y., Zhang, S., Wang, B., Liu, X., Wu, J., Williams, P.H. (2014). The bumble bees of North China (Apidae, Bombus Latreille). *Zootaxa* 3830, 1−89.  Ji, R., Xie, BY., Yang, GH., Li, D. From introduced species to invasive species-a case study on the Italian bee *Apis mellifera* L. *Chinese Journal of Ecology*, 2003, 22(5):70-73.  Sanpa, S. and Chantawannakul, P. (2009): Survey of six bee viruses by using RT-PCR in Northern Thailand. *Journal of Invertebrate Pathology* 100 (2), 116-119.  Yang, GH. Harm of introducing the western honeybee *Apis mellifera* L. to the Chinese honeybee *Apis cerana* and it ecological impact. *Acta Entomologica Sinica*, 2005, 48(3):401-406. [In Chinese with English summary.] | |
| 47 | The spread of *Apis cerana* | *Apis cerana* is the sister species of the honey bee *Apis mellifera*. Ecologically the two species occupy very similar niches and historically they have occurred in separate geographic ranges, but as a consequences of human activity this is changing. *A. mellifera* has been introduced across much of the range of *A. cerana. A. cerana* is spreading across the range of *A. mellifera*. Recently *A. cerana* has become established in Papua New Guinea, The Soloman Islands and Australia. The two species do not coexist well, but it is not clear which wins out of the competition. *A. mellifera* drones do not seem to differentiate between *A. mellifera* and *A. cerana* queens and hence load *A. cerana* queens with incompatible sperm. Conversely diseases and pests have moved from *A. cerana* to *A. mellifera* – the most famous being *Varroa* and *Nosema cerana*. Across much of China the introduction and maintenance of *A. mellifera* has led to a decline in *A. cerana* populations. In the Soloman islands, however, it seems *A. cerana* has completely outcompeted *A. mellifera* since the accidental introduction of *A. cerana* there in 2003. We don’t know why *A. mellifera* sometimes outcompetes *A. cerana* and why sometimes the opposite happens. *A. cerana* arrived in Australia as recently as 2012. It remains unclear how fast it is spreading or how *A. mellifera* will react to *A. cerana* in Australia. | Somerville D. 2011 Asian bees. PrimeFact 1093 NSW Government Industry and Investment | |
| 48 | Use of managed bees to reduce human-wildlife conflict | In Africa, elephants, where they still occur, range widely outside of protected areas. Crops represent a rich and easy source of forage, and there is often human-wildlife conflict between elephants and crop farmers. Electric fences are too expensive for most crop farmers and elephants are not only large and intimidating, but also intelligent, so difficult to control. In East Africa, research has now shown that honey bee colonies attached to fences can control crop raiding by elephants (<http://www.zoo.ox.ac.uk/impact/elephants_and_bees>). Elephants were found to react rapidly to the sound of aggressive bees, retreating away from the sound. A study carried out over two years on 34 farms using bee hives attached to fences found that of 45 elephant raids recorded, there was only one incident of an elephant crossing a bee hive fence (<http://www.zoo.ox.ac.uk/impact/elephants_and_bees>). There is now interest in ascertaining if southern African elephants react as their East African counterparts, and if honey bees can be used in this regard to prevent problems in northern KwaZulu Natal (South African National Biodiversity Institute 2015), and perhaps other locations, like Zimbabwe and Mozambique. Similarly, there is interest as to whether Asian honey bees (*Apis cerana*), although less aggressive than African honey bees, may be comparable deterrents in Asia. Similarly, indigenous managed bees may also be useful in reducing other human-wildlife conflict in other areas. | South African National Biodiversity Institute. 2015. *Towards a national strategy for sustaining managed pollintion in South Africa*. Pretoria. | |
| 49 | Substances that affect pollinator memory | There is some selection for pollinators being able to associate floral scent with forage reward, which increases their foraging efficiency, in turn improving pollination and seed set, through enhance pollinator fidelity. A recent study (Wright et al. 2013) found that compounds in floral nectar, like caffeine, found in both coffee and citrus nectar, can affect pollinators’ behaviour. At natural concentrations, caffeine increased worker honeybee’s ability to remember the association between floral scent and nectar reward, because caffeine acts as an adenosine receptor antagonist, which affected neurons associated with learning and memory (Wright et al. 2013). It is also known that glycophosate (used for weed control), when used in the field at recommended concentrations led to impaired short term memory in honey bees, although no effect on foraging behaviour was found (Herbert et al. 2014). The authors speculate that successful foraging bees could import nectar containing traces of glycophosate into hives, ultimately distributing it to the rest of the colony, which could have long term consequences on colony performance (Herbert et al. 2014). There is potential to research the effect of subsidising bee forage with other compounds beneficial to memory and foraging performance, and trying to reduce the presence of those that impair performance. | Herbert, L.H., Vazquez, D.E., Arenas, A., & Farina, W.M. 2014. Effects of field-realistic doses of glyphosate on honeybee appetitive behaviour. *The Journal of experimental biology*. doi: 10.1242/jeb.109520  Wright, G. , Baker, D.D., Palmer, M.J., Stabler, D., Mustard, J., Power, E.F., Borland, M., & Stevenson, P.C. 2013. Caffeine in floral nectar enhances a pollinator’s memory of reward. *Science* 339: 1202–4. | |
| 50 | National and global monitoring: limited progress without them | For some taxa, and some geographic regions, we have high quality data on the status and trends of pollinators. For instance, we know about shifts in the diversity and distributions of bees and hoverflies in Europe and North America, and the abundance of butterflies in a few European countries; however, for the rest of the world we have almost no quantitative information. Therefore, even though we know that many of the drivers of pollinator declines are prevalent outside of Europe and North America, we have no idea about the status and trends of pollinators in these regions. Further, even where monitoring schemes are currently in operation they are not designed to be able to tell us whether mitigation actions (e.g. nature reserves, agri-environment schemes, changes in pesticide use) are having any measurable effect, plus we are failing to measure other critical aspects of pollinators such as pollination services to crops an wild plants. Without national and global monitoring it is impossible to identify those pollinators, crops and wild plants most at risk, and in what areas, or assess whether any of our interventions are effective.  Generic designs have been proposed to allow governments and scientists to monitor pollinator trends across different scales, monitoring schemes for some species are already running, and new tools, such as high throughput DNA metagenomics are able to provide efficient and quick ways of identifying species. All these components could be brought together to develop nested monitoring schemes capturing the most important aspects of pollinator biodiversity at local to national to global scales. The challenge is how to integrate and implement (e.g. through citizen or professional scientists) in a way that links outputs to actions of stakeholders, such as policy makers, conservationists and farmers. If successfully implemented, monitoring schemes could provide: an early warning system for loss of species of conservation concern or key crop pollinators; a way to better understand how putative drivers are linked to pollinator health and populations; and targeting of where interventions are most needed. | LeBuhn G. et al. (2013) Detecting insect pollinator declines on regional and global scales. *Conservation Biology* 27: 113–120  Tang M. et al. (2015) High-throughput monitoring of wild bee diversity and abundance via mitogenomics. Methods in Ecology and Evolution DOI: 10.1111/2041-210X.12416 | |
| 51 | Altered evolutionary trajectories in plants and pollinators | The movement of plants and animals around the global, along with the extinction of other plants and pollinators, has arguably led to changes in all plant-pollinator communities (Kearn et al 1998; Eckert et al 2010). The loss of pollinators can lead to severe pollen limitation with population level effects (Pattemore & Anderson 2013; Anderson et al 2011), while introductions of new pollinators can dramatically change selection pressures on floral traits (Galen 1996). Floral traits can evolve rapidly in response to changes in their pollinators or other selection pressures (Galen 1996; Raguso & Pichersky 1995), and major shifts in effective pollinator taxa can be induced by single mutations (Bradshaw & Schemske 2003). It is likely, therefore, that human-induced changes in the composition of plant-pollinator communities is altering the trajectory of floral and pollination evolution. As yet, the consequences of these shifts are unknown. | Kearn C.A., Inouye D.W., Waser N.M. (1998) Endangered Mutualisms: The Conservation of Plant-Pollinator Interactions. *Annual Review of Ecology and Systematics*, 29:83-112.  Eckert C.G., Kalisz S., Geber M.A. et al. (2010) Plant mating systems in a changing world. *Trends in Ecology & Evolution*, 25:35-43.  Pattemore D.E., Anderson S.H. (2013) Severe pollen limitation in populations of the New Zealand shrub *Alseuosmia macrophylla* (Alseuosmiaceae) can be attributed to the loss of pollinating bird species. *Austral Ecology*, 38:95-102  Anderson S.H., Kelly D., Ladley J.J., Molloy S., Terry J. (2011) Cascading effects of bird functional extinction reduce pollination and plant density. *Science*, 331:1068-1071.  Galen, C (1996) Rates of Floral Evolution: Adaptation to bumblebee pollination in an alpine wildflower, *Polemonium viscosum*. *Evolution*, 50:120-125.  Floral volatiles from *Clarkia breweri* and *C. concinna* (Onagraceae): recent evolution of floral scent and moth pollination. *Plant Systematics and Evolution*, 194:55-67  Bradshaw Jr. H. D., Schemske, D.W. (2003) Allele substitution at a flower colour locus produces a pollinator shift in monkey flowers. *Nature*, 426:176-178. | |
| 52 | Environmental and ecological effect of dams | Dams have played an important role in human development throughout the world, providing water, controlling floods, irrigating crops, facilitating navigation, creating recreational opportunities, and generating electricity (Rosenberg et al. 2000; WCD 2000), but the dam and associated environmental alterations may result in a wide range of regional changes in terrestrial and aquatic biodiversity, as well as in ecosystem structure and functioning (Milliman 1997). For examples, all along the Colorado River in the US — one of the most regulated bodies of water in the world — the habitat for fish, birds, and other wildlife is near collapse (Kelly 2006). It has been reported that inundation and resettlement would affect a total of 22 vegetation types, including four wood communities, nine shrub communities, and nine grass communities (Tian et al. 2007). So the environmental impacts of the dam on biodiversity and ecological processes have raised concerns to scholars in related fields worldwide, and France and the United States, have suspended their construction, in some cases, have even initiated their demolition (WCD 2000; McCormack 2001), however, Until recently, large dams are still perceived as a symbol of progress in hydraulic engineering and economic development in some counties, such as three gorges dam (TGD) were constructed in the Yangtze River basin in China, three gorges project lies in a region which is considered to be one of the three richest flora centres in China (Ying 2001) and also one of the 25 biodiversity hotspots in the world (Myers et al. 2000). TGD construction which associated environmental alterations may result in a wide range of regional changes in terrestrial and aquatic biodiversity, as well as in ecosystem structure and functioning. One obvious impact of the TGD is the unprecedented change in biodiversity due to reservoir environments, and would affect the wildlife, especially pollinators in the future (Li et al., 2013). | Li, KF. Zhu, C., Wu, L. Problems caused by the Three Gorges Dam construction in the Yangtze River basin: A review. *Environ. Rev*. 21: 127–135 (2013) dx.doi.org/10.1139/er-2012-0051  McCormack, G. 2001. Water margins: Competing paradigms in China. *Critical Asian Studies*, 33(1): 5–30. doi:10.1080/14672710122114.  Milliman, J.D. 1997. Blessed dams or damned dams? *Nature*, 386: 325–327. doi: 10.1038/386325a0.  Myers, N., Mittermeier, R.A., Mittermeier, C.G., da Fonseca, G.A.B., and Kent, J. 2000. Biodiversity hotsposts for conservation priorities. *Nature*, 403: 853–858. doi:10.1038/35002501. PMID:10706275.  Rosenberg, D.M., McCully, P., and Pringle, C.M. 2000. Global-scale environmental effects of hydrological alterations: Introduction. *BioScicence*, 50(9): 746–751. doi:10.1641/0006-3568(2000)050[0746:GSEEOH]2.0.CO;2.  Tian, Z., Chen, W., Zhao, C., Chen, Y., and Zheng, B. 2007. Plant biodiversity and its conservation strategy in inundation and resettlement districts of the Yangtze Three Gorges, China. *Acta Ecological Sinica*, 27: 3110–3118. [In Chinese with English summary.] doi:10.1016/S1872-2032(07)60065-1.  Ying, T. 2001. Species diversity and distribution pattern of seed plants in China. *Biodivers. Sci*. 9: 393–398. [In Chinese with English summary.]  WCD (World Commission on Dams). 2000. Dams and Development: A New Framework for Decision Making. Earthscan Publications, London. | |
| 53 | The bee band-wagon | With increased media, public and political concern about bee decline, there is an increase in the uptake of bee-keeping and bee-friendly planting schemes in both urban and agricultural settings (Carvell et al. 2007, Blackmore and Goulson 2014). This may benefit managed species (in particular, *Apis mellifera* and other managed honeybee species) and common, generalist species (such as short-tongued bumblebees or anthrophilic hoverflies, with a broad ecological niche), but there may be little benefit to other pollinators and pollination services. For example, inappropriate “wildflower” planting may provide inadequate or even toxic nutritional resources, and exacerbate non-native plant species invasion (with knock-on consequences for native vegetation and pollinators). “Bee hotels” have become common as a means for promoting bees, when in fact they only benefit a fraction of cavity nesting species, and can be more often colonised by native wasps and non-native bees (MacIvor and Packer 2015). | Blackmore, L.M. & Goulson, D. (2014) Evaluating the effectiveness of wildflower seed mixes for boosting floral diversity and bumblebee and hoverfly abundance in urban areas. *Insect Conservation and Diversity*. **7,** 480-484.  Carvell, C., Meek, W.R., Pywell, R.F., Goulson, D. & Nowakowski, M. (2007) Comparing the efficacy of agri-environment schemes to enhance bumble bee abundance and diversity on arable field margins. *Journal of Applied Ecology,* **44,** 29-40  MacIvor, J.S. & Packer, L. (2015) ‘Bee hotels’ as tools for native pollinator conservation: A premature verdict? *PLoS ONE,* **10,** e0122126. | |
| 54 | The Media | Misreporting, misrepresenting the facts, muddying the waters, looking for a story… confusion over what is driving pollinator decline, what people can do to help and what it means for service provision does not help science, crop production or conservation. People may get “pollinator fatigue” and resources could be diverted to alternative areas before we make any real progress. |  | |
| 55 | Focus on technology and commercialisation in science funding | with pollinator decline, more technologies for artificial pollination are being developed including pollen dusting (<http://sciencelearn.org.nz/Contexts/Pollination/Sci-Media/Video/Artificial-pollination>) and bee robots (e.g. the Harvard Robobee: Ma al. 2013). It is possible that research funding for innovation and technology which is commercialisable will be given priority over ecology and conservation, which could hold back research into pollinator and pollination service decline. | Ma K, Chirarattananon P, Fuller SB, Wood RJ (2013) Controlled Flight of a Biologically Inspired, Insect-Scale Robot. *Science* 340(6132):603-7 | |
| 56 | Destruction of roosting sites for pollinating bats worldwide | Bats are important pollinators and seed-disperser in tropical forests and to some desert ecosystems, but also to cultivated plants such as agave and durian (Bumrungsri et al. 2009). The absence of bats would lead to a gradual breakdown in the ecosystem as plants would have difficulties to reproduce and provide food and protection to wildlife species (BCI 2015a). However, human activities such as mining or direct cave destruction, blocking of cave entrances impeding bat emergence and changing the cave topoclimate, guano extraction and disturbance of the animals by collection of nests of cave-nesting birds and tourism is increasingly endangering caves or disturbing bat roosting sites in many parts of the world, such as Brazil, Guatemala, Thailand and Indonesia (Trajano 1995, Cajas et al. 2015). In opposition to that, abandoned mines have been used by bats but they need t be protected from destruction or having their entrances closed (BCI 2015b). Although many nectarivorous bat species do not depend exclusively on caves, the great majority of individuals roost on them and these sites are essential for population stability. As human activities are expected to expand over tropical forests and karstic areas around the world in the near future, the reduction of present or potential roosting sites can reduce bat populations in environments where they are vital pollinators. | Bat Conservation International. 2015a. Why bats are important. http://www.batcon.org/why-bats/bats-are/bats-are-important. Accessed 25 August 2015.  Bat Conservation International. 2015b. Cave & mine destruction. <http://www.batcon.org/our-work/regions/usa-canada/address-serious-threats/cave-mine-destruction-landing>. Accessed 25 August 2015.  Bumrungsri S., Sripaoraya E., Chongsiri T., Sridith K. and P. A. Racey. 2009. The pollination ecology of durian ( *Durio zibethinus*, Bombacaceae) in southern Thailand. *Journal of Tropical Ecology*, 25: 85-92. doi:10.1017/S0266467408005531  Cajas C., J.O., José Luis Echeverría T., J.L. and L. Trujillo. 2015. Murciélagos del “Parque Nacional Cuevas del Silvino”, Izabal, Guatemala. Programa para la Conservación de los Murciélagos de Guatemala (PCMG). Ciudad de Guatemala. 10p.  Trajano, E. 1995. Protecting caves for the bats or bats for the caves? *Chiroptera Neotropical*, 1(2): 19-21 | |
| 57 | Reproductive division of labor and susceptibility to stressors | Despite being sheltered by workers, queens in the social insects may nevertheless be affected by stressors, which is crucial because the role of queens in social insect colony survival is indispensable. Indeed, reports of poor queen failure are accumulating amongst beekeepers and the usually long lived queens can be exposed to stressors over long time periods (e.g. pesticides) and may succumb to them throughout the year (e.g. queens in *Apis mellifera*). However, the vast majority of studies has focused on workers and very little is known about the impact of stressors on queens. | Williams GR, Troxler A, Retschnig G, Roth K, Yañez O, Shutler D, Neumann P, Gauthier L (2015) Neonicotinoid pesticides severely affect honey bee queens. *Scientific Reports*, in press | |
| 58 | Gene drive technology to eradicate invasive pollinators | Gene Drive technology has been discussed in the academic literature extensively over the past two years and its potential use to eradicate invasive species was discussed in a 2012 horizon scan (Sutherland *et al*. 2014). The methodology works by identifying a gene that can be altered to make a species less resistant to pesticides or less able to reproduce, and this modification will be passed down generations until the species is eradicated. Eradication of invasive *Bombus terrestris* in Argentina might be necessary to prevent extinction of *Bombus dahlbomii* , for example (Morales *et al*. 2013; Schmid-Hempel *et al*, 2014) . However, whilst the technique can be applied to any species, recent work suggests the results are not predictable and the possibility of the mutated genes being passed to native populations, or mutating further to produce unpredictable results, remains a risk. Since 2013, Cas9 or CRISPR is the gene-editing technique that has proved most successful. Because of the technique’s ability to target specific genes to mutate, not only can it be used to spread traits it can also be used to prevent cells from mutating and forming resistance. | | <http://www.sciencemag.org/content/345/6197/626>  <http://en.wikipedia.org/wiki/Gene_drive>  <http://news.nationalgeographic.com/news/2014/07/140717-gene-drives-invasive-species-insects-disease-science-environment/>  Sutherland, W. J., Aveling, R., Brooks, T. M., Clout, M., Dicks, L. V., Fellman, L., . . . Watkinson, A. R. (2014). A horizon scan of global conservation issues for 2014. *Trends in ecology & evolution* 29(1), 15-22. Retrieved from <http://linkinghub.elsevier.com/retrieve/pii/S0169534713002772>  Morales, C. L., Arbetman, M. P., Cameron, S. A., & Aizen, M. A. (2013). Rapid ecological replacement of a native bumble bee by invasive species. *Frontiers in Ecology and the Environmen*t, 11(10), 529-534. doi:10.1890/120321  Schmid-Hempel, R., Eckhardt, M., Goulson, D., Heinzmann, D., Lange, C., Plischuk, S., . . . Schmid-Hempel, P. (2014). The invasion of southern South America by imported bumblebees and associated parasites. *Journal of Animal Ecology*, 83(4), 823-837. doi:10.1111/1365-2656.12185 |
| 59 | Impacts of IPBES pollinators assessment | Pollinators are the subject of one of the first thematic assessments to be conducted by the Intergovernmental Platform on Biodiversity and Ecosystem Services. This will be published in February 2016. Its impacts are difficult to predict, but there is a potential for substantial policy changes in response. This include either increases or decreases in action on pollinators, or research investments, nationally and internationally. | http://www.ipbes.net/index.php/40-work-programme/experts/517-deliverable-3a | |
| 60 | Extinctions of flower-visiting birds | A range of plants, especially in the tropics, are particularly adapted to bird pollination. A recent analysis (Regan et al. 2015) found that of 1,089 bird species known to visit flowers (10% of all known bird species!), an average of 1 species per year is moving up a Red List category (i.e., closer to extinction). If many flower-visiting birds are declining in numbers, what is the current, and likely future impact of this on tropical plant communities? | Regan *et al*. (2015) Global trends in the status of bird and mammal pollinators. *Conservation Letters* http://onlinelibrary.wiley.com/doi/10.1111/conl.12162/abstract | |
